# Fatty Acid Oxidation Supports Lymph Node Metastasis of Cervical Cancer via Acetyl‐CoA‐Mediated Stemness

**DOI:** 10.1002/advs.202308422

**Published:** 2024-03-23

**Authors:** Li Yuan, Hongye Jiang, Yan Jia, Yuandong Liao, Caixia Shao, Yijia Zhou, Jiaying Li, Yan Liao, Hua Huang, Yuwen Pan, Weijia Wen, Xueyuan Zhao, Linna Chen, Xu Jing, Chaoyun Pan, Wei Wang, Shuzhong Yao, Chunyu Zhang

**Affiliations:** ^1^ Department of Obstetrics and Gynecology The First Affiliated Hospital Sun Yat‐sen University Guangzhou 510080 China; ^2^ Guangdong Provincial Clinical Research Center for Obstetrical and Gynecological Diseases Guangzhou 510080 China; ^3^ Department of Microbiology Tumor and Cell Biology Karolinska Institute Stockholm 17165 Sweden; ^4^ Department of Biochemistry and Molecular Biology Zhongshan School of Medicine Sun Yat‐sen University Guangzhou 510080 China

**Keywords:** cervical cancer, fatty acid oxidation, lymph node metastasis, stemness

## Abstract

Accumulating evidence indicates that metabolic reprogramming of cancer cells supports the energy and metabolic demands during tumor metastasis. However, the metabolic alterations underlying lymph node metastasis (LNM) of cervical cancer (CCa) have not been well recognized. In the present study, it is found that lymphatic metastatic CCa cells have reduced dependency on glucose and glycolysis but increased fatty acid oxidation (FAO). Inhibition of carnitine palmitoyl transferase 1A (CPT1A) significantly compromises palmitate‐induced cell stemness. Mechanistically, FAO‐derived acetyl‐CoA enhances H3K27 acetylation (H3K27Ac) modification level in the promoter of stemness genes, increasing stemness and nodal metastasis in the lipid‐rich nodal environment. Genetic and pharmacological loss of CPT1A function markedly suppresses the metastatic colonization of CCa cells in tumor‐draining lymph nodes. Together, these findings propose an effective method of cancer therapy by targeting FAO in patients with CCa and lymph node metastasis.

## Graphical abstract

Metabolic reprogramming of cancer cells supports the energy and metabolic demands during tumor metastasis. This study reveals that inhibition of carnitine palmitoyl transferase 1A significantly compromises palmitate‐induced tumor cell stemness and lymph node metastasis. Fatty acid oxidation‐derived acetyl‐CoA enhances H3K27 acetylation modification level in the promoter of stemness genes, thereby increasing tumor cells' metastatic colonization in the lipid‐rich nodal environment.

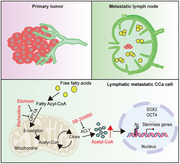

## 1 Introduction

Cervical cancer (CCa) is one of the most common female cancers worldwide, with 604000 new cases and 342000 deaths in 2020.^[^ ^1^ ^]^ Due to the special anatomy of the pelvis, cervical cancer cells mainly spread to the pelvic and para‐aortic lymph nodes through lymphatic vessels.^[^ ^2^ ^]^ Patients with CCa and lymph node metastasis (LNM) have a higher recurrence rate and a lower 5‐year overall survival rate.^[^ ^3^, ^4^ ^]^ The 2018 International Federation of Gynecology and Obstetrics (FIGO) stage also recommended that patients with CCa and LNM should be diagnosed with stage IIIc, regardless of primary tumor size and extension,^[^ ^5^ ^]^ suggesting that LNM status has a pivotal role in the prognosis of patients with CCa. Therefore, it is necessary to elucidate the underlying molecular mechanism of LNM and explore promising therapeutic targets for CCa.

Metabolic reprogramming is a hallmark of malignant tumors, allowing tumor cells to overcome the metabolic challenges in the different stages of metastasis.^[^ ^6^, ^7^ ^]^ A key concept linked to metabolic plasticity is nutrient availability in metastatic sites,^[^ ^8^, ^9^ ^]^ which may help explain why tumor cells prefer metastasis to specific organs rather than others. Specific nutrients such as glucose, glutamine, pyruvate, and fatty acids support the proliferation of metastasizing cells when they seed and colonize in a secondary site.^[^ ^8^, ^10^, ^11^ ^]^ Recently, fatty acid oxidation (FAO) and lipid metabolism have received considerable attention for their critical role in the progression of various types of cancers, including cervical cancer.^[^ ^12^, ^13^, ^14^ ^]^ For example, solute carrier organic anion transporter family member 1B3 (SLCO1B3) was reported to promote FAO of high‐grade serous ovarian cancer (HGSOC), promoting its metastasis.^[^ ^15^ ^]^ Concordantly, our previous study found that fatty acid‐binding protein 5 (FABP5) is significantly upregulated in CCa with LNM, and FABP5 overexpression markedly promotes LNM by activating the NF‐κB signaling pathway.^[^ ^14^ ^]^ However, whether FAO is increased in LNM and how it occurs in CCa remains elusive.

In the present study, we uncovered a potential mechanism of metastasis in CCa mediated by FAO. We identified enhanced FAO as a novel metabolic hallmark of metastatic CCa cells, and both genetic loss and pharmacological inhibition of CPT1A compromised LNM. Specifically, FAO controls the activation of an H3K27ac‐stemness axis that is essential for CCa cell colonization and growth in lymph nodes. Together, our study indicates that CPT1A and the FAO are the central regulators of metabolic plasticity, supporting CCa cell stemness and LNM. These findings uncover a potential target to prevent LNM of CCa.

## 2 Results

### 2.1 Metastatic CCa Cells Display Increased FAO in Lymph Nodes

**Figure 1:**
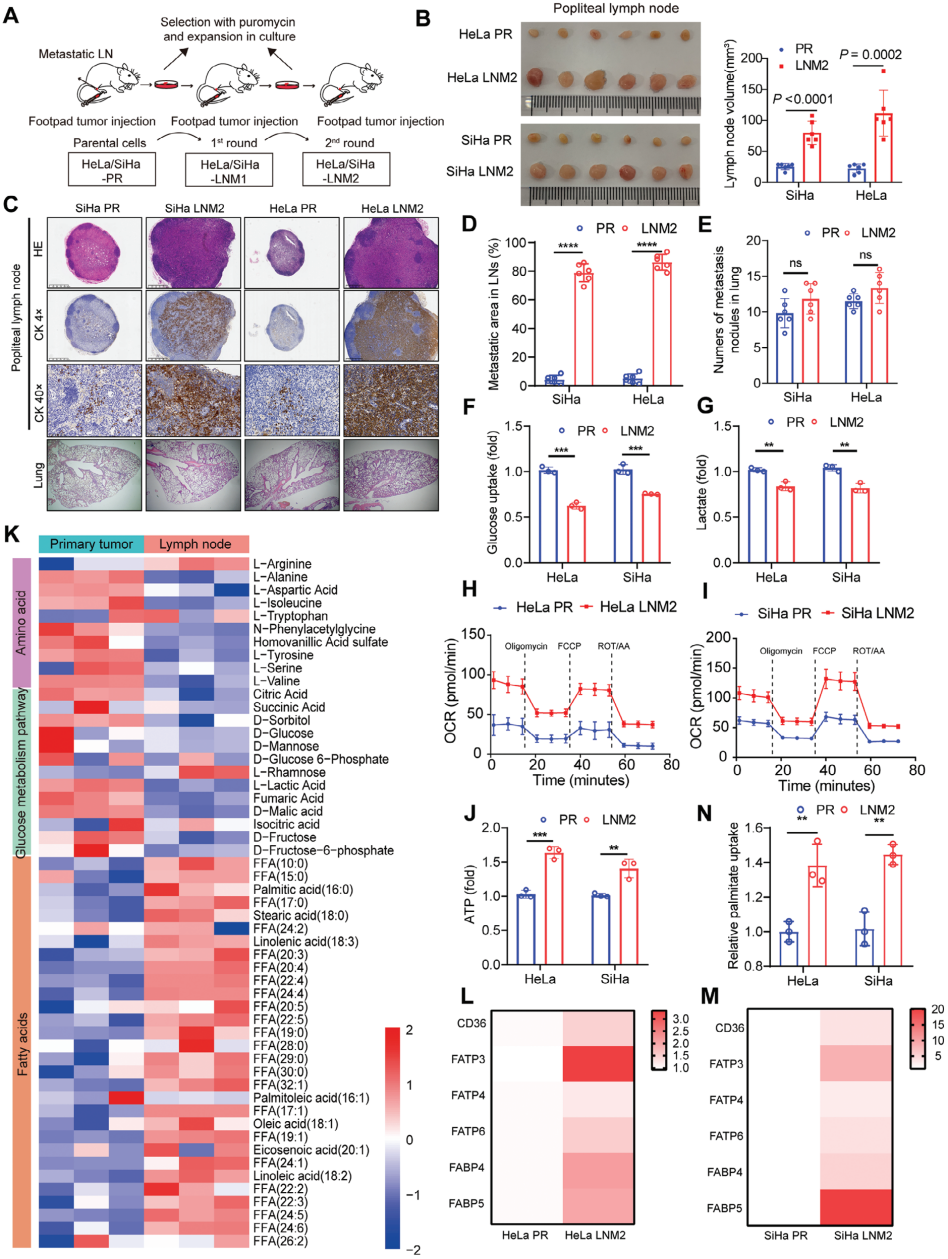
Metastatic cervical cancer cells in lymph nodes display increased fatty acid oxidation. A) Schematic diagram of the screening of highly metastatic cervical cancer cells. B) Representative images and the LNs volume of popliteal LNs in different groups (*n* = 6). C) Representative images of HE of lungs and immunostaining of pan‐cytokeratin of popliteal LNs to indicate metastatic CCa cells (*n* = 6). D) Quantification of the metastasis area in LNs in the indicated groups (*n* = 6). E) Quantification of the number of metastasis nodules in the lung in the indicated groups (*n* = 6). F,G) Glucose Uptake‐Glo assay and lactate assay in the indicated groups. H,I) OCR measured in HeLa PR, HeLa LNM2, SiHa PR, and SiHa LNM2 cells (*n* = 3 in H, *n* = 4 in (I)). J) ATP generation and palmitate uptake in the indicated groups (*n* = 3). K) Comparison of metabolome profiles between footpads and lymph nodes (*n* = 3). L,M) Heatmap showed the expression level of fatty acid uptake and transport‐associated genes (*n* = 3). N) Relative palmitate uptake in CCa PR and LNM2 cells (*n* = 3). Error bars represent the means ± SD. Statistical analyses were performed using a two‐tailed Student's *t*‐test. ^**^ *p* < 0.01; ^***^ *p* < 0.001; and ^****^ *p* < 0.0001.

To better understand the potential mechanism of LNM of CCa, we successfully established highly lymph node‐metastatic CCa cell lines, confirmed by in vivo LNM screening. Among several cervical cancer cell lines, HeLa and SiHa cell lines were chosen since they are HPV‐positive and are derived from primary CCa but not from metastatic organs like omentum or intestines.^[^ ^16^ ^]^ We used the nude mice popliteal LNM model to simulate the pelvic linear drainage of the lymphatic system in CCa.^[^ ^17^ ^]^ Briefly, the HeLa and SiHa cells were inoculated into the footpads of nude mice. After 6 weeks, metastatic popliteal lymph nodes were dissected and CCa cells colonized in lymph nodes were screened by the addition of puromycin (2 µg mL^−1^) expanded in culture, and re‐injected into the footpad of mice for the second round (**Figure** **1**A). After two rounds of in vivo selection, the lymph node‐metastatic subpopulation of HeLa and SiHa cells was established and named HeLa‐LNM2 and SiHa‐LNM2. We assessed the LNM capacity of HeLa‐LNM2 and SiHa‐LNM2 compared with their parental counterparts (HeLa‐PR and SiHa‐PR) in vivo. The results showed that the volume of the popliteal lymph nodes was significantly larger in the HeLa‐LNM2 and SiHa‐LNM2 groups than in the parental cell groups (Figure 1B). Moreover, immunostaining of pan‐Cytokeratin was performed to mark CCa cells, showing that LNM2 cells had an obviously increased area of LNM compared with the corresponding parental cell lines (Figure 1C,D). Besides, the murine lung metastasis model revealed that HeLa‐LNM2 and SiHa‐LNM2 cells did not have enhanced lung metastasis after tail vein injection (Figure 1E), suggesting that HeLa‐LNM2 and SiHa‐LNM2 cells specially metastasize to lymph nodes.

We compared metabolic differences between LNM2 and PR cells to assess the key metabolic alterations contributing to the LNM of CCa. Aerobic glycolysis, which increases the metabolism of glucose to lactate, is widely associated with tumor growth and metastasis. However, we found that both the glucose uptake and lactate generation were significantly reduced in HeLa‐LNM2 and SiHa‐LNM2 cells compared with their parental counterparts (HeLa‐PR and SiHa‐PR cells) (Figure 1F,G). These findings indicate that lymph node‐metastatic CCa cells use an alternative metabolic pathway rather than glycolysis to maintain their survival and growth in lymph nodes. In addition to glucose metabolism, tumor cells can utilize other metabolic pathways, like FAO, to maintain their survival during metastasis. We measured oxygen consumption rate (OCR) in lymph node‐metastatic CCa cells using a Seahorse extracellular flux analyzer, and the results showed that the basal and maximal respiration were both enhanced in HeLa‐LNM2 and SiHa‐LNM2 cells compared with HeLa‐PR and SiHa‐PR cells (Figure 1H,I). Moreover, an increased ATP production was found in metastatic CCa cells compared with parental cells (Figure 1J), indicating that FAO was the main source of energy in lymph node‐metastatic CCa cells.

Metabolic environment changes in lymph nodes might also affect the metabolism of adjacent cancer cells. To explore this possibility, we conducted metabolomics profiling to investigate whether the metabolic alternations of CCa cells metastasizing to lymph nodes were attributed to the nutrient availability in lymph nodes. Our metabolomics analysis revealed that lymph nodes have a higher abundance of fatty acids than the primary tumor, whereas metabolites related to glucose or amino acid metabolism showed an opposite trend (Figure 1K), which was consistent with previous findings.^[^ ^8^ ^]^ Interestingly, we found that genes related to fatty acid uptake and transportation were highly expressed in nodal metastatic CCa cells (Figure 1L,M). In fact, we found a higher palmitate uptake rate in HeLa‐LNM2 and SiHa‐LNM2 cells than in their parental cells when supplemented with extra [9,10‐3H(N)]‐palmitate (Figure 1N). These findings suggest that metastatic CCa cells might rely on lipid uptake from their surrounding environment and FAO for better adaption and colonization in lymph nodes. Together, we demonstrated that lymph node metastatic CCa cells have increased FAO.

### 2.2 CPT1A Was Upregulated in Lymph Node Metastatic CCa Cells

**Figure 2:**
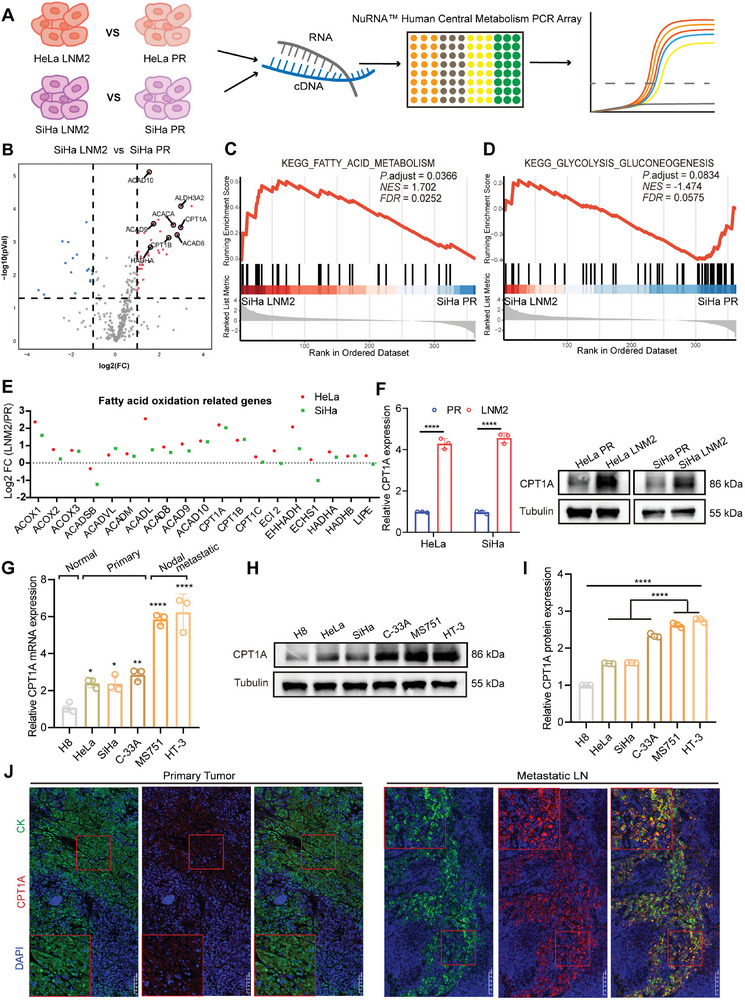
CPT1A was upregulated in metastatic cervical cancer cells. A) Schematic diagram of the NuRNA Human Central Metabolism PCR Array. B) Differentially expressed transcripts involved in cell metabolism are depicted in a volcano plot. The black dotted lines indicate a fold‐change threshold of 2, and the *p* value cutoff (*p*  = 0.05). C,D) Gene set enrichment analyses of pathways in Figure 2b. The FAO pathway was significantly activated and the glycolysis‐gluconeogenesis pathway showed no significant difference. E) The relative expression of FAO‐associated genes in HeLa LNM2 and SiHa LNM2 cells compared with HeLa PR and SiHa PR cells. F) The mRNA and protein levels of CPT1A were upregulated in CCa metastatic cells (*n* = 3). G–I) Relative mRNA levels and protein levels of CPT1A in a variety of human cervical cancer cell lines (*n* = 3). J) IF analyses of CPT1A and pan‐cytokeratin in primary tumor and LNs metastatic site of tumor‐bearing nude mice. Error bars represent the means ± SD. Statistical analyses were performed using a two‐tailed Student's *t*‐test. ^*^ *p* < 0.05; ^**^ *p* < 0.01; and ^****^ *p* < 0.0001.

Lipid metabolic reprogramming might be a major energy pathway contributing to the continuous tumor growth of cancer cells in lymph nodes. Thus, we focused on the details of FAO pathway. We conducted a metabolomics PCR assay on lymph node metastatic CCa cells and their parental cells to gain a deeper understanding of LNM‐related metabolic alterations at the transcriptional level (**Figure** **2**A). Compared with parental cells, lymphatic metastatic CCa cells showed significantly increased expression of FAO‐related genes (Figure 2B; Figure S1A, Supporting Information). Gene set enrichment analyses (GSEA) demonstrated that differentially expressed metabolic enzymes were enriched in FAO, whereas glycolysis was not activated (Figures 2C,D; S1B,C, Supporting Information). We used quantitative reverse‐transcriptase polymerase chain reaction (RT‐qPCR) to validate the results of metabolic PCR. FAO‐related genes were mostly upregulated in lymph node‐metastatic CCa cells compared with their parental counterparts, and CPT1A, the rate‐limiting enzyme of FAO, had the highest expression in both HeLa‐LNM2 and SiHa‐LNM2 cells (Figure 2E). We also confirmed the enhancement of CPT1A expression at mRNA and protein levels in HeLa‐LNM2 and SiHa‐LNM2 cells (Figure 2F). Besides, CPT1A was highly expressed in CCa cell lines relative to normal cervix cell line H8. Moreover, CPT1A was more abundant in lymph node‐metastatic cell lines HT‐3 and MS751 than in HeLa, SiHa, and C33A cell lines derived from the primary tumor (Figure 2G–I).^[^ ^18^ ^]^ The nude mice lymph node metastasis model indicated that lymph node‐metastatic CCa cells had an increased CPT1A expression compared with corresponding primary tumors (Figure 2J). Thus, our findings indicated that CPT1A was upregulated in lymph node‐metastatic CCa cells.

### 2.3 Genetic or Pharmacological Blockade of FAO Inhibits LNM of CCa Cells

**Figure 3:**
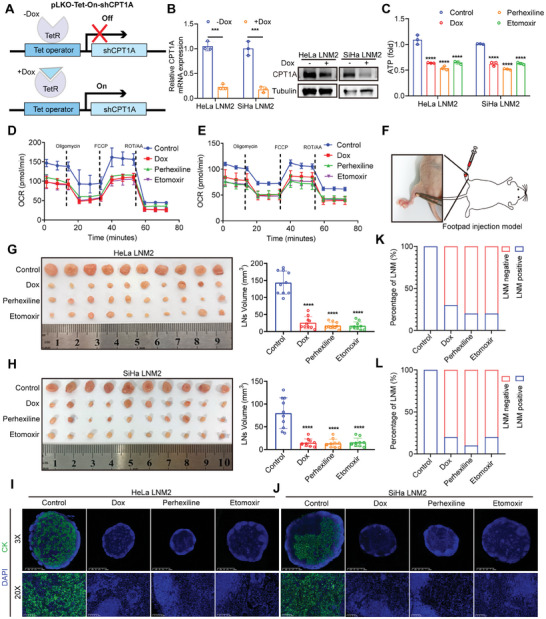
Blocking fatty acid oxidation by CPT1A inhibition impaired lymph node metastasis of cervical cancer cells. A,B) The schematic illustration of the doxycycline‐inducible knockdown of CPT1A (A), and the CPT1A expression levels treated with or without doxycycline (B). C—E) ATP generation (C) and OCR (D, E) measured in indicated groups. F) Schematic diagram of the footpad injection model. G,H) Representative images and the LNs volume of popliteal LNs in different groups (*n* = 10). I,J) Representative images of immunofluorescence staining of pan‐cytokeratin of popliteal LNs. K,L) Percentage of lymph node metastasis in the indicated groups. Error bars represent the means ± SD. Statistical analyses were performed using a two‐tailed Student's *t*‐test. ^***^ *p* < 0.001 and ^****^ *p *< 0.0001.

To investigate the effect of FAO‐related metabolic reprogramming on LNM of CCa, we pharmacologically inhibited CPT1A or genetically silenced it as a rate‐limiting enzyme of FAO.^[^ ^19^ ^]^ We used the doxycycline‐inducible system for CPT1A knockdown, and doxycycline (Dox) successfully induced CPT1A depletion in CCa cells (**Figure** **3**A,B). Meanwhile, etomoxir and perhexiline, two inhibitors of CPT1, were used for the pharmacological blockade of FAO. Significant reduction in ATP levels was found in Dox‐treated, etomoxir‐treated, and perhexiline‐treated SiHa/HeLa‐LNM2 cells compared to control cells (Figure 3C). The result of OCR assay demonstrated that inhibition of CPT1A by Dox, Etomoxir, and Perhexiline impaired the basal and maximum respiration of HeLa‐LNM2 and SiHa‐LNM2 cells (Figure 3D,E). Then, we assessed LNM formation in vivo by injecting CCa cells into the footpad of nude mice (Figure 3F). The results showed that both genetic (Dox‐induced shCPT1A) and pharmacological (perhexiline or etomoxir treatment) inhibition of CPT1A markedly reduced LNM, evidenced by smaller lymph node volume and lower metastatic percentage (Figure 3G–L). Although shCPT1A, perhexiline, and etomoxir suppressed the LNM of CCa cells, the proliferation and apoptosis rate of primary tumors in the footpad were not affected after these treatments (Figure S2A,B, Supporting Information). These findings suggest that FAO is essential for LNM of CCa but does not contribute to the growth of primary tumors.

**Figure 4:**
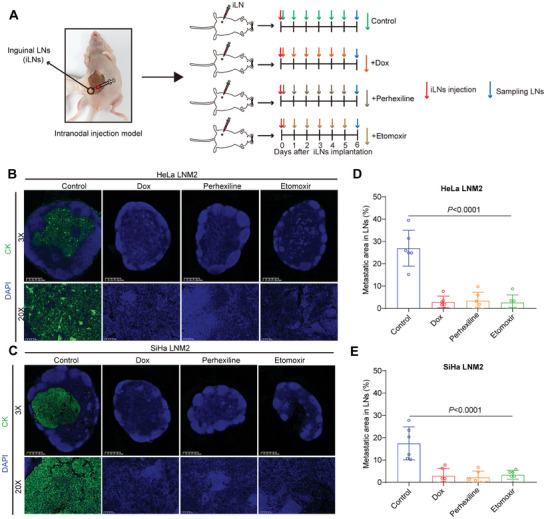
Blocking fatty acid oxidation by CPT1A inhibition impaired lymph node colonization of CCa cells. A) Schematic diagram depicting generation of a direct implantation of CCa cells into LNs. After implantation of HeLa LNM2 and SiHa LNM2 cells into inguinal LNs, PBS, doxycycline diet (2 mg mL^−1^), perhexiline (3 mg kg^−1^) or etomoxir (40 mg kg^−1^) was treated daily for 7 days before harvesting the LNs. B–E) Representative IF images of inguinal LNs (B,C) and comparison of metastatic tumor area between PBS, Dox, perhexiline, or etomoxir‐treated groups (*n* = 6 for each). Error bars represent the means ± SD. Statistical analyses were performed using a two‐tailed Student's *t*‐test. ^****^ *p *< 0.0001.

Colonization is the last and most important phase of metastatic formation.^[^ ^20^, ^21^ ^]^ To investigate whether FAO influenced lymph node colonization of CCa cells, we directly implanted CCa cells into the inguinal LNs of mice. After 6 days of injection, mice were sacrificed, their inguinal LNs were harvested, and survived CCa cells were detected by immunofluorescence staining (**Figure** **4**A). CCa cells were recognized by human cytokeratin (Pan‐CK) antibody (Figure 4B,C). We also found that CK^+^ area in inguinal LNs was smaller after CPT1A depletion or inhibition (Figure 4D,E), implying the decreased number of survived CCa cells and impaired metastatic colonization of LNM2 cells in lymph nodes. These findings support that FAO plays an important role in the metastatic colonization of CCa cells. These results were consistent with previous findings that lymph nodes are a relatively lipid‐rich environment (Figure 1K), in which CCa cells prefer to use free fatty acids and FAO to survive. These data support that FAO inhibition is an effective treatment strategy for CCa with LNM.

Since fatty acid oxidation could promote lymph node metastasis and colonization of CCa cells, we further explored whether a high‐fat diet (HFD) could increase the metastatic ability of CCa cells in the nude mice model. Notably, mice fed with a HFD developed larger lymph node volume compared with a normal diet (ND) (Figure S3A,B, Supporting Information). Furthermore, we found an increased metastatic area of LNs in HFD groups (Figure S3C,D, Supporting Information), which is in accordance with a previous study that HFD specifically boosted the metastatic potential of CD36^+^ metastasis‐initiating cells in oral cancer.^[^ ^22^ ^]^ Together, these results suggest that HFD contributes to lymph node metastasis, which may be attributed to the increased fatty acids level and nutrient availability in lymph nodes.

Tumor‐associated macrophages are abundant in the tumor microenvironment.^[^ ^23^, ^24^ ^]^ To exclude the involvement of tumor‐associated macrophages (TAM) in FAO‐induced lymph node metastasis, we treated tumor‐bearing mice with Clodronate, a liposome‐based agent to deplete macrophages. Clodronate treatment effectively depleted macrophages in the CCa tumor (Figure S4A,B, Supporting Information). Despite the ablation of TAM cells, the lymphatic metastasis of vehicle and clodronate‐treated tumors showed no difference in both groups. Meanwhile, compared with the Control group, pharmacological inhibition of CPT1A with etomoxir inhibited highly metastatic cancer cells lymph node metastasis in both vehicle and clodronate‐treated groups (Figure S4C–E, Supporting Information). Since macrophages in the tumor microenvironment often exhibit an M2 phenotypic transition, which has been associated with tumor metastasis, we compared the total and M2 phenotypes of macrophages in primary tumors and lymph nodes with or without CPT1A inhibition. The loss function of CPT1A had no significant effect on F4/80+ macrophage infiltration and CD206^+^ M2 macrophage population (Figure S5A,B, Supporting Information). These findings suggest that macrophage polarization is less likely to contribute to the FAO‐mediated lymph node metastasis of CCa in our experimental settings.

### 2.4 FAO Enhances the Stemness of CCa Cells

**Figure 5:**
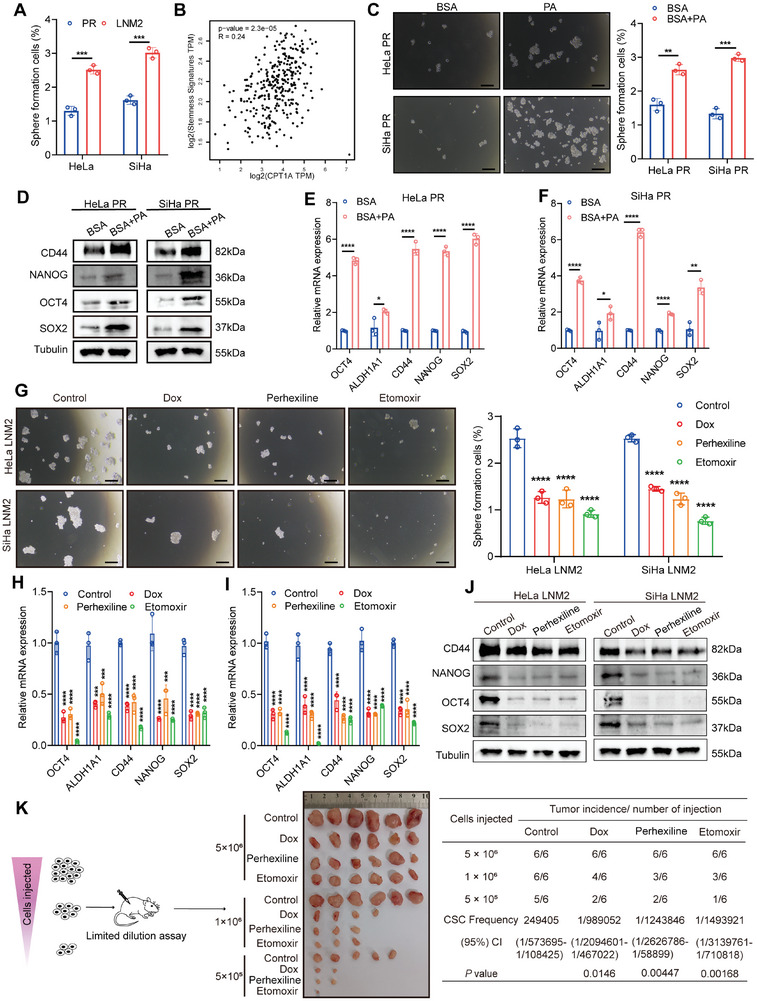
Fatty acid oxidation enhanced the stemness of CCa cells. A) Sphere formation rate of CCa PR cells and LNM2 cells. B) Pearson correlation of CPT1A expression and stemness signatures (SOX2, OCT4, NANOG, CD44, ALDH1A1) in cervical cancer in the GEPIA datasets. C) Sphere formation assay and sphere formation rate of CCa cells treated with BSA or PA. Bar = 150 µm. D) The protein expression levels of stemness‐related genes in CCa cells under different treatments. E,F) Relative mRNA levels of stemness‐related genes in CCa cells under different treatments. G) Sphere formation assay and sphere formation rate of CCa cells under different treatments. Bar = 150 µm. H–J) Relative mRNA and protein levels of stemness‐related genes in different groups. K) In vivo limiting dilution xenograft formation after subcutaneous injection with CCa cells in different gradients. Each experiment was performed at least three times independently. Error bars represent the means ± SD. Statistical analyses were performed using a two‐tailed Student's *t*‐test. ^*^ *p *< 0.05; ^**^ *p* < 0.01; ^***^ *p* < 0.001; and ^****^ *p* < 0.0001.

Stemness is an important property for tumor metastasis and colonization.^[^ ^25^ ^]^ To illuminate the underlying mechanisms, we measured the stemness of CCa LNM2 cells and PR cells by a series of in vitro assays. Both HeLa LNM2 cells and SiHa LNM2 cells showed higher sphere formation efficiency than their parental counterparts (**Figure** **5**A; Figure S6A, Supporting Information). Additionally, we measured the expression of pluripotency‐related transcription factors SOX2, OCT4, and NANOG, and CCa stem cell marker CD44 in LNM2 and PR CCa cells. We found higher expression of stemness signature genes in LNM2 cells (Figure S6B–D, Supporting Information), suggesting the enhanced stemness properties of metastatic CCa cells. The ALDEFLUOR assay also revealed a higher percentage of ALDH^+^ cells in HeLa LNM2 and SiHa LNM2 cells compared with their parental cells (Figure S6E,F, Supporting Information). Moreover, using the Gene Expression Profiling Interactive Analysis (GEPIA) database (http://gepia.cancer‐pku.cn/), we detected a significantly positive correlation between CPT1A expression and stemness signatures at the transcriptome level (Figure 5B), indicating that FAO might regulate tumor stemness. All these findings supported the fact that metastatic CCa cells had enhanced tumor stemness properties which is necessary for LNM.

Then, we explored whether palmitate supplementation, which mimics the lipid‐rich microenvironment, can induce the stemness signature of CCa cells. We found an increased sphere formation efficiency and stemness gene expression in PA treatment groups (Figure 5C–F). ALDEFLUOR assay also showed that the ALDH‐positive ratio in CCa cells increased after PA treatment (Figure S7A,B, Supporting Information). In contrast, we found that the enhanced sphere formation ability of SiHa/HeLa‐LNM2 cells was significantly attenuated by CPT1A inhibition (Figure 5G). Similarly, inhibition of CPT1A dramatically suppressed the increased mRNA levels of stemness‐related genes and the percentage of ALDH^+^ subpopulations in lymph node‐metastatic CCa cells (Figure 5H–J; Figure S7C–E, Supporting Information). Moreover, an in vivo limited dilution assay using different dosages of SiHa LNM2 cells revealed that the initiation capacity of LNM2 cells was significantly repressed by inhibiting FAO (Figure 5K), suggesting the pivotal role of CPT1A‐mediated FAO in maintaining cancer stem cell (CSCs)‐like properties of CCa cells during the lymph node colonization and metastasis.

### 2.5 FAO Promotes Acetyl‐CoA Mediated H3K27 Acetylation of the Stemness‐Related Gene Promoters

**Figure 6:**
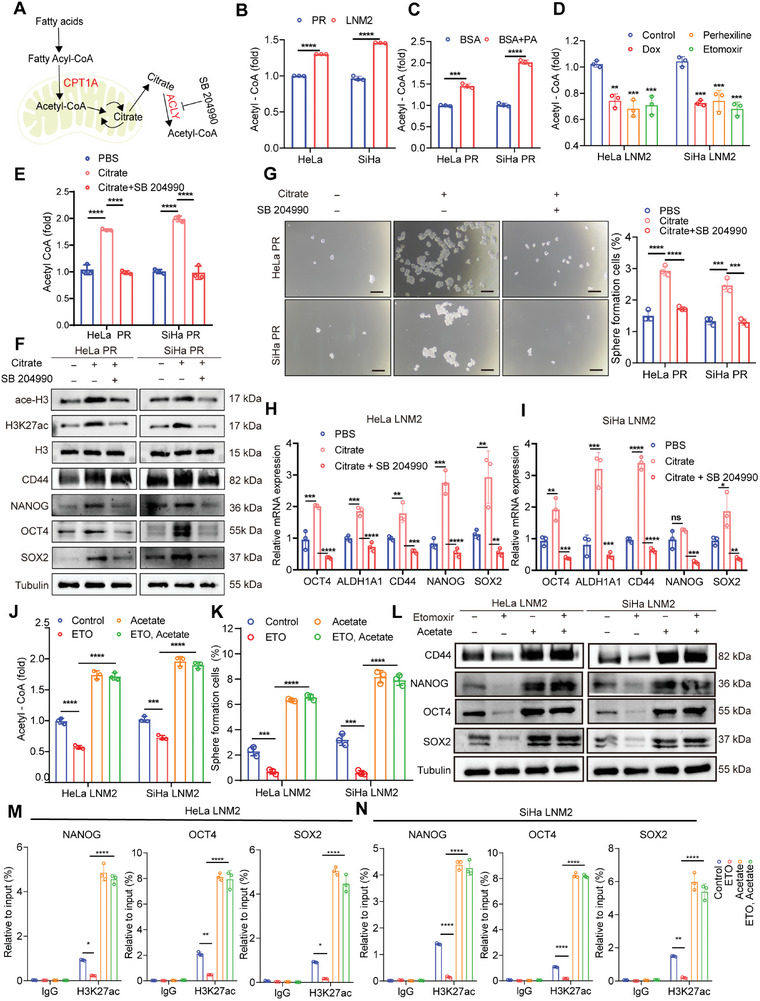
Fatty acid oxidation enhanced the stemness of CCa cells via H3K27 acetylation. A) Schematic diagram depicting the process of fatty acid oxidation‐derived acetyl‐CoA. B–E) Acetyl‐CoA level in the indicated groups. F) Relative protein levels of stemness‐related genes and acetylation level of H3 and H3K27ac under different treatments. G) Sphere formation assay and sphere formation rate of CCa cells in indicated groups. Bar = 150 µm. H,I) Relative mRNA levels of stemness‐related genes in indicated groups. J) Acetyl‐CoA level upon etomoxir treatment, with and without acetate supplementation. K) Sphere formation rate of CCa cells in indicated groups. L) Protein level of stemness‐related genes in indicated groups. M,N) The interaction of H3K27Ac and NANOG, OCT4, and SOX2 promotors in CCa cells under different treatments was assessed by ChIP‐PCR. IgG was used as a negative control. Each experiment was performed at least three times independently. Error bars represent the means ± SD. Statistical analyses were performed using a two‐tailed Student's *t*‐test. ns no significance; ^*^ *p* < 0.05; ^**^ *p* < 0.01; ^***^ *p* < 0.001; and ^****^ *p *< 0.0001.

To uncover how FAO enhanced the stemness of metastatic CCa cells, we analyzed the effects of FAO on gene expression. CPT1A contributes to the transport of long‐chain fatty acyl‐CoAs into mitochondria for subsequent FAO and acetyl‐CoA production (**Figure** **6**A). As an important metabolite derived from various metabolic processes, acetyl‐CoA has been reported to influence cancer stem‐like properties in liver cancer and breast cancer.^[^ ^26^, ^27^ ^]^ Therefore, we investigated whether acetyl‐CoA played a role in FAO‐induced stem‐like properties of metastatic CCa cells. We found that acetyl‐CoA abundance was higher in metastatic CCa cells and CCa cells supplemented with extra palmitate compared with their parental counterparts. Genetic and pharmacological inhibition of CPT1A significantly decreased acetyl‐CoA abundance in HeLa/SiHa‐LNM2 cells (Figure 6B–D).

Acetyl‐CoA is a key regulator of histone acetylation, which is sensitive to nuclear acetyl‐CoA levels.^[^ ^28^, ^29^, ^30^ ^]^ Moreover, our results unveiled that FAO augmentation or inhibition could increase or decrease acetyl‐CoA levels. Subsequently, we investigated whether acetyl‐CoA‐mediated histone acetylation linked FAO to tumor stemness in CCa cells. Strikingly, we found that total acetylation of histone H3 and acetylation of histone H3 at lysine 27 (H3K27ac is an important marker of active gene promoter^[^ ^31^, ^32^ ^]^) was increased in CCa cells upon palmitate treatment, whereas CPT1A inhibition led to an opposite effect (Figure S8A,B, Supporting Information). Besides, citrate supplementation increased acetyl‐CoA and H3K27ac levels in HeLa PR and SiHa PR cells. On the contrary, inhibition of ATP citrate lyase, which converts citrate into acetyl‐CoA in the cytosol and/or nuclear, with SB204990 reduced acetyl‐CoA and histone acetylation levels to a similar extent as CCa PR cells (Figure 6E). Consistent with FAO‐mediated histone acetylation, sphere formation capacity, and stemness signature genes were enhanced by citrate treatment, whereas SB204990, an ATP citrate lyase inhibitor reversed such effects (Figure 6F–I). Collectively, these results suggest that CPT1A‐derived acetyl‐CoA can enhance CCa cell stemness via acetyl‐CoA‐mediated histone acetylation.

Acetate can be converted into acetyl‐CoA and replenish acetyl‐CoA pools.^[^ ^33^ ^]^ To validate the role of acetyl‐CoA in CPT1A‐induced cancer stem‐like properties, we investigated whether acetate supplementation could reverse the effect of CPT1A inhibition on HeLa and SiHa cells. Notably, acetate supplementation increased cellular H3K27 acetylation level and tumor cell stemness properties in a dose‐dependent manner (Figure S8C–E, Supporting Information). Moreover, treating CCa cells with acetate effectively increased the cellular concentration of acetyl‐CoA and H3K27 acetylation levels in etomoxir‐treated SiHa/HeLa‐LNM2 cells (Figure 6J). Similarly, the sphere formation and expression of stemness‐related genes were also recovered after acetate supplementation (Figure 6K,L; Figure S8F, Supporting Information), suggesting that FAO modulated cancer stemness of CCa cells in an acetyl‐CoA‐dependent manner. Furthermore, we conducted chromatin immunoprecipitation (ChIP) with an anti‐H3K27ac antibody followed by qPCR (ChIP‐qPCR) using primers targeting the promoter of each stemness‐related gene. The results showed that H3K27 acetylation on the promoter region of NANOG, SOX2, and OCT4 was decreased after etomoxir treatment, while treatment with acetate reversed these effects (Figure 6M,N). These observations indicate that increased H3K27ac acetylation induces the transcription of stemness‐related genes in lymph node‐metastatic CCa cells. Therefore, we concluded that FAO could lead to the accumulation of acetyl‐CoA, which enhances H3K27ac modification in the promoter regions of stemness‐related genes and increases their expression to support the colonization of CCa cells in lymph nodes.

### 2.6 Increased CPT1A Expression Was Correlated with the Malignant Phenotype of CCa Cells

**Figure 7:**
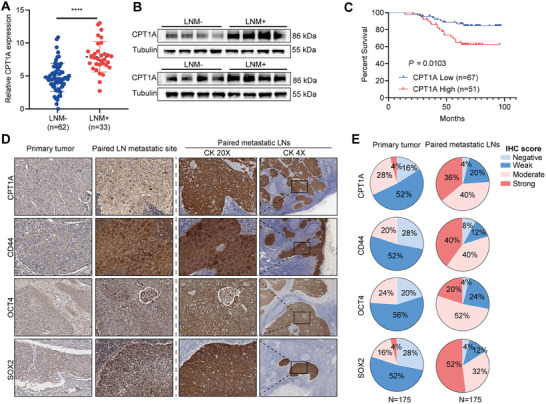
CPT1A was upregulated in LN‐metastatic cervical cancer cells and reversely correlated with the patient's survival. A) RT‐qPCR analysis of CPT1A expression in CCa samples with or without lymph node metastasis. B) Relative protein level of CPT1A in CCa samples (*n* = 8) with or without lymph node metastasis (*n* = 8). C) Kaplan–Meier analysis showed a negative correlation between CPT1A expression levels and overall survival. D) IHC analyses of CPT1A, CD44, OCT4, and SOX2 proteins in primary tumor and paired metastatic lymph node. E) The distribution of these proteins staining between the primary tumor and metastatic site was compared, *n* = 175. A value was calculated using a two‐tailed Student's *t*‐test; C value was calculated using Kaplan–Meier Plotter in patients with cervical cancer.

To further elaborate the clinical impact of CPT1A in patients with CCa, we detected the mRNA expression levels of CPT1A in CCa tissues with (*n* = 33) and without (*n* = 62) LNM. We found that patients with LNM had higher CPT1A expression in the primary tumors compared with those who did not develop LNM (**Figure** **7**A,B). Moreover, we found that enhanced CPT1A expression in primary tumors of CCa patients was significantly associated with increased lymph node metastasis and decreased overall survival (Figure 7C; Table S1, Supporting Information). Furthermore, higher expression of CPT1A and stemness markers (CD44, OCT4, and SOX2) was also observed in the metastatic lymph nodes compared with paired primary CCa tumors (Figures 7D,E; S9A, Supporting Information). These data suggest that CPT1A‐mediated FAO supports LNM in patients with CCa, and targeting FAO might block this process.

## 3 Discussion

Here, we analyzed nutrient availability in lymph nodes and found that fatty acids, such as palmitate, are highly enriched in the nodal environment. Moreover, we found that the lipid‐rich environment is functionally linked to enhanced FAO and the stemness of metastatic CCa cells. We found that CPT1A was significantly upregulated in LN‐metastatic CCa cells compared with their parental counterparts, and acetyl‐CoA was produced through FAO‐regulated acetylation levels of H3K27 in the promoter regions of CSC‐related genes. Inhibition of CPT1A, either in a genetic or pharmacological manner, effectively blocked LNM of CCa, providing a potential therapeutic target for CCa patients with LNM.

Metabolic reprogramming is critically involved in metastatic colonization, and different metabolic phenotypes of cancer cells arise because of the nutrient availability in their environment.^[^ ^34^ ^]^ For instance, pyruvate is abundant in the lung niche, where metastatic breast cancer cells use pyruvate to drive collagen‐based remodeling of the extracellular matrix and produce α‐ketoglutarate.^[^ ^35^ ^]^ The latter then promotes collagen hydroxylation and the growth of breast cancer‐derived lung metastases.^[^ ^35^ ^]^ Changes in nucleotide metabolism have also been observed during metastatic colonization of breast cancer, and inhibition of de novo synthesis of nucleotides markedly suppressed the pulmonary metastasis of breast cancer cells.^[^ ^10^ ^]^ Another study discovered that several bioactive bile acids highly accumulate in the metastatic lymph nodes of melanoma, and these bile acids activated Yes‐associated protein (YAP) in tumor cells, subsequently upregulating genes in the FAO signaling pathway.^[^ ^8^ ^]^ Moreover, increasing dietary fats, such as palmitic acid, was sufficient to promote metastasis in melanoma and oral carcinomas, and inhibition of fatty acid uptake significantly impaired the metastasis of various tumors.^[^ ^22^, ^36^ ^]^ However, how metabolism remodeling facilitates CCa cell colonization in lymph nodes and the underlying molecular mechanisms have not been fully elucidated. In the present study, we established a LNM model by injecting CCa cells into the footpad and screening highly metastatic cells in mice. Using the metabolomics analysis, we found that lymph node‐metastatic CCa cells had enhanced fatty acid metabolism compared with their parental counterparts. Interestingly, we also found that a series of genes involved in fatty acid uptake and transportation were also activated in LN‐metastatic CCa cells. For example, CD36, the best characterized fatty acid transporters, has been regarded as a marker of lymph node‐metastatic cells, and those cells displayed an increased FAO signature.^[^ ^22^ ^]^ These data further supported the importance of fatty acid uptake and oxidation for LNM. In fact, we confirmed that targeting CPT1A can effectively block LNM of CCa cells via both genetic and pharmacological loss‐of‐function approaches without significant influence on the growth of primary tumors. The footpad was a relatively lipid‐poor microenvironment. Thus, FAO might not be the main metabolic pathway for primary CCa cells, and disrupting FAO had a negligible effect on CCa cell proliferation and apoptosis. Therefore, our present data complements the role of FAO, particularly palmitate oxidation, in tumor cell metastasis.

Maintenance of stem‐like phenotypes is a prerequisite for metastatic colonization.^[^ ^37^, ^38^, ^39^ ^]^ However, the association between metabolism and stemness remains uncovered. In breast cancer, CD96‐mediated FAO regulates cancer stemness and chemoresistance.^[^ ^40^ ^]^ In our study, we found that metastatic CCa cells have enhanced stemness properties, evidenced by enhanced sphere formation and upregulation of stemness signature genes. In addition, activation of FAO by palmitate acid enhanced the stemness of CCa cells, whereas CPT1A inhibition demonstrated the opposite effect. Based on in vitro and in vivo experiments, we concluded that FAO was responsible for enhanced stemness of LN‐metastatic CCa cells.

Besides providing ATP, NADH, NADPH, and FADH2 as the source of energy, FAO generates acetyl‐CoA as the main intermediate product. Recent studies have shown the potential implication of FAO in protein acetylation.^[^ ^41^, ^42^ ^]^ Acetyl‐CoA produced by FAO not only fulfills the mitochondrial acetyl‐CoA pool but is also exported to the cytosol by citrate carriers in the form of citrate.^[^ ^43^, ^44^ ^]^ Citrate is then catabolized to acetyl‐CoA by ACLY and replenishes the cytosolic and nuclear acetyl‐CoA pool.^[^ ^45^, ^46^ ^]^ Emerging evidence reveals that acetyl‐CoA produced through FAO is essential for the acetylation of mitochondrial and cytosolic proteins, and almost up to 90% of the total acetyl‐CoA content for nuclear histone acetylation is produced via FAO.^[^ ^47^ ^]^ Here, we proposed that acetyl‐CoA derived from FAO promoted CCa stemness properties by increasing H3K27 acetylation on stemness‐related gene expression, such as NANOG, OCT4, and SOX2. We also demonstrated that exogenous citrate and/or acetate could promote H3K27Ac and stemness of CCa cells after FAO inhibition. Thus, FAO is critically involved in stemness properties by regulating histone acetylation, linking metabolism, and epigenetic modification in CCa cells. Our finding supports nutrient‐dependent regulation of histone acetylation in LN‐metastatic CCa cells. However, as FAO replenishes the total acetyl‐CoA pools, whether the acetylation of LNM‐related mitochondrial or cytosolic proteins is affected by FAO needs further investigation.

**Figure 8:**
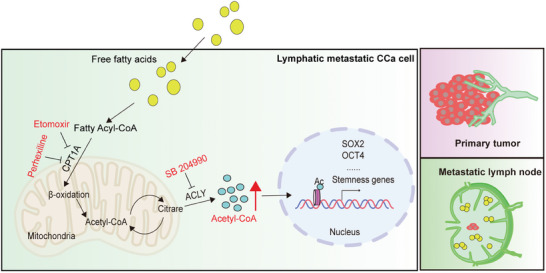
A schematic diagram of the mechanism. FAO‐derived acetyl‐CoA enhanced the H3K27 acetylation modification level in the promoter of stemness genes, increasing stemness and nodal metastasis in the lipid‐rich nodal environment.

In conclusion, our experimental and clinical data support the role of CPT1A‐mediated FAO in promoting LNM of CCa. Mechanistic analysis indicated that acetyl‐CoA produced via FAO serves as a substrate for histone acetylation of stemness‐related genes, supporting the metastatic growth of CCa cells in the lipid‐rich environment of lymph nodes (**Figure** **8**). Thus, CPT1A may be a novel prognostic marker for lymph node‐metastatic CCa cells, and FAO inhibitors may effectively inhibit LNM. Based on our findings, it will be interesting to design clinical trials measuring the effects of FAO inhibitors on the lymph node metastasis of human cancer.

## 4 Experimental Section

### Clinical Specimens

CCa tissues and matched lymph node tissues were obtained from patients who underwent gynecological surgery between 2011 and 2017 at the First Affiliated Hospital of Sun Yat‐sen University (Guangzhou, China). None of the enrolled CCa patients had radiotherapy or chemotherapy prior to surgery and were at stages Ia2 to IIa2 with regular follow‐up dates; they had also undergone radical hysterectomy and lymphadenectomy. This study was approved by the Ethical Review Committee of the First Affiliated Hospital of Sun Yat‐sen University (approved number: IIT‐2022‐205). Patient studies were conducted in accordance with the Declaration of Helsinki. All human tumor tissues were obtained with written informed consent from patients or their guardians prior to participation in the study.

### Cell Culture

Human CCa cell lines (HeLa, SiHa, C‐33A, MS751, HT‐3) and a normal cervical cell H8 were purchased from the American Type Culture Collection (ATCC, USA). All CCa cell lines were cultured in DMEM with 10% fetal bovine serum (Gibco, USA) and 0.5% penicillin/streptomycin (Gibco, China). Cells were cultured in a humid atmosphere with 5% CO2 at 37 °C. In 2021, all of the cell lines used were tested for authenticity by short tandem repeat (STR) genotyping; the cell lines were also screened for mycoplasma contamination (e‐Myco Mycoplasma PCR Detection Kit; iNtRON).

### Generation of the LN Metastatic HeLa and SiHa Cells (HeLa LNM2 and SiHa LNM2)

The LNs metastasis‐prone adapted HeLa and SiHa cells were generated as previously reported.^[^ ^8^ ^]^ Briefly, HeLa and SiHa cells pre‐transfected the plasmid with purinomycin resistance. Then, the metastatic popliteal LNs from implantation of HeLa and SiHa cells into footpads of nude mice were harvested and digested, and the tumor cells were selected in vitro by the addition of puromycin (2 µg mL^−1^). Next, the expanded tumor cells were re‐implanted into the nude mice footpads. After two rounds of in vivo selection, high metastatic HeLa and SiHa subpopulations (named HeLa LNM2 and SiHa LNM2) were established.

### Animal Models

All animal procedures were approved by the Sun Yat‐sen University Animal Care Committee (approved number: SYSU‐IACUC‐2022‐001362). Female BALB/c nude mice (4–6 weeks of age, 18–20 g) were purchased from the Experimental Animal Center of Sun Yat‐sen University and raised under SPF conditions. HFD experiments were performed by feeding mice a 60/Fat Rodent Diet (Research diets, D12492) for 2 weeks before tumor cells were injected into the footpad of nude mice. For the footpad implantation model, CCa cells (2 × 10^6^/50 µL per mouse) were implanted subcutaneously into the footpad region of the female nude. Four weeks later, popliteal LNs were dissected for further experiments and analyses. For FAO inhibition, etomoxir (40 mg kg^−1^ of body weight) or perhexiline (3 mg kg^−1^ of body weight) was injected daily subcutaneously to the anterolateral side of the mouse leg, or doxycycline diet was given from the second week of implantation of CCa cells. For depletion of tumor macrophages, 200 µL Clophosome (FormuMax, F70101C‐N,) was used and intravenously injected every 4th day. For direct implantation of CCa cells into lymph nodes, 1 × 10^5^ CCa cells in 10 µL PBS were directly injected into inguinal LNs of female nude mice using a 30 gauge syringe. The inguinal LNs were sampled on day 6 after implantation. At the experimental endpoint, nude mice were anesthetized and sacrificed; their lymph nodes were removed, measured, and embedded with paraffin for immunofluorescence. The lymph node volumes were calculated using the following formula: Volume(mm^3^) = (length[mm])×(width[mm])^2^×0.52. Simple randomization was used to allocate mice into different groups and no blinding was done.

### Plasmid Construction and Transfection, Lentivirus Production and Transduction

According to the manufacturer's protocols, plasmid transfection was carried out using X‐tremeGENE HP DNA Transfection Reagent (Roche, Germany). To produce the lentiviral particles, lentiX‐293T cells were cultured to reach 70% confluence in a 10 cm dish and then co‐transfected with an expression vector of interest the psPAX2 vector (7.5 µg), the pMD2.G vector (2.5 µg), and the vector of interest (10 µg). After 48 h, viral particles were collected from the supernatant of cultures lenti‐293T cells and passed through Millipore Millex‐GP Filter Unit with 0.45 µm pore size to remove cell debris. To generate Dox‐inducible CPT1A depletion cell lines, cell lines were infected with lentivirus encoding pLKO‐Tet‐On‐shCPT1A, and then selected with puromycin (2 µg mL^−1^) for 5 days. Doxycycline hyclate (D9891; Sigma, St.Louis, MO, USA) was dissolved in ddH_2_O (2 mg mL^−1^) (in vivo experiment) or added to the culture medium at a final concentration of 10 µg mL^−1^ in order to induce depletion of the CPT1A.

### RNA Extraction and Quantitative Real‐Time PCR

Total RNA was extracted from the cultured cells and tumors using the SteadyPure Universal RNA Extraction Kit (ACCURATE BIOTECHNOLOGY(Hunan) CO., LTD, Changsha, China) in accordance with the manufacturer's instructions. Then, quantitative real‐time PCR was (qRT‐PCR) performed as described previously. Actin was used as internal normalization for qRT‐PCR experiments in this study and the results were presented as relative expressions to control. All primers were synthesized by GENEWIZ (Suzhou, China), and primer sequences are given in Table S2 (Supporting Information).

### Western Blotting

For western blotting analysis, harvested cells were lysed on ice in RIPA lysis buffer supplemented with protease and phosphatase inhibitors (Cell Signaling Technology, #5872). The lysates were centrifuged for 15 min at 4 °C, 13 000 g. The protein concentration of the supernatants was quantitated using the detergent‐insensitive Pierce BCA protein assay kit (Thermo Scientific, 23 227). Loading buffer (Thermo Fisher, NP0007) was added to total protein lysates, and samples were denatured at 97 °C for 5 min. Aliquots of each protein lysate (25 µg) were subjected to SDS–polyacrylamide gel electrophoresis. After electrophoresis, proteins were transferred to nitrocellulose membranes and blocked for 60 min with 5% BSA. Primary antibodies were incubated at 4 °C for overnight. After washes, membranes were incubated with secondary antibodies for 60 min at room temperature (RT). Target proteins were detected using an ECL western blot detection solution (Millipore, WBKLS0500). Primary antibodies used for western blotting in this study are given in Table S3 (Supporting Information).

### Immunohistochemistry (IHC) and Immunofluorescence Staining

For IHC, tissues were dewaxed in xylene and then rehydrated in ethanol with gradient concentrations. After PBS rinsing, the sections were incubated in 0.01 mol L^−1^ sodium citrate buffer (pH 6.0) and antigen retrieval was performed at a high temperature. After blocking the endogenous peroxidases in 3% H_2_O_2_ at room temperature for 15 min, the tissues were blocked with 5% goat serum in PBST and then incubated with corresponding primary antibodies at 4 °C overnight. After washes with PBS, these tissues were incubated with horseradish peroxidase‐ (HRP‐) labeled rabbit goat at RT for 60 min. Positive expressions were determined by 3,3′‐diaminobenzidine (DAB) staining solution. The intensity of IHC staining (I) was scored as 0 (negative), 1 (weak), 2 (medium), or 3 (strong) grades. The percentage of positively stained cells (P) was scored as 1 (≤25%), 2 (26–50%), 3 (51–75%), or 4 (≥75%) grades. IHC score (Q) = P × I, in which *P* was the percentage of positive cells and I was the intensity of IHC staining. For IF, tissues were dewaxed in xylene and then rehydrated in ethanol with gradient concentrations. After PBS rinsing, the sections were incubated in 0.01 mol L^−1^ sodium citrate buffer (pH 6.0) and antigen retrieval was performed at a high temperature and washed with PBS. Next, the tissues were blocked with 5% donkey serum and then incubated at 4 °C overnight with corresponding primary antibodies. After several washes, the tissues were incubated for 60 min at RT with the corresponding secondary antibodies. Nuclei were stained with 4,6‐diamidino‐2‐phenylindole (DAPI, Invitrogen). Then, the immunofluorescent images were acquired using an LSM880 confocal microscope (Carl Zeiss). Antibodies used in this study are listed in Table S3 (Supporting Information).

### Limiting Dilution Analysis

SiHa LNM2 cells with different treatment conditions were resuspended in PBS at limiting dilution conditions (5 × 10^5^, 1 × 10^6^, and 5 × 10^6^ per 75 µL) and injected into subcutaneous female nude mice, respectively. The stem cell frequency was calculated with ELDA software (http://bioinf.wehi.edu.au/software/elda/).

### Sphere Formation Assay

Cells were resuspended placed into the 6‐well ultralow plate in stem cell medium, serum‐free DMEM/F12 media (ThermoFisher, C11330500BT) supplemented with 20 ng mL^−1^ hEGF (PeproTech, AF‐100‐15‐100), 20 ng mL^−1^ hFGF recombinant human protein (PeproTech, 100–18B‐100), and 2% B27 supplement (GIBCO, 17504‐044) for 5–7 days at 37 °C with 5% CO_2_. The stem cell media medium was supplemented every 2 days and the spheres were counted.

### Chemical Reagents

Palmitic acid (Catalog #P9767), citrate (Catalog #791 741), and perhexiline (Catalog #SML0120) were obtained from Thermo Fisher. SB 204 990 (Catalog #B5782‐1) was obtained from APExBIO. Etomoxir (Catalog #S8244) was obtained from Selleck Chemicals. For all in vitro assays, perhexiline (10 µm), etomoxir (100 µm), palmitic acid (100 µm), citrate (1 mm), and SB 204 990 (25 µm) were co‐cultured with CCa cells. For in vivo experiments, perhexiline (3 mg kg^−1^) and etomoxir (40 mg kg^−1^) were injected into nude mice, respectively.

### Microarray Analysis

The NuRNA Human Central Metabolism PCR array (Arraystar Inc., MD, USA) was used to identify dysregulated transcripts in HeLa LNM2 and SiHa LNM2 cells compared with HeLa PR and SiHa PR cells.

### Chromatin Immunoprecipitation Assay

The chromatin immunoprecipitation (ChIP) assay was performed using the EZ‐Chip Kit (Millipore, Catalog # 17–10086) following the manufacturer's instructions as previously reported.^[^ ^17^ ^]^

### Acetyl‐CoA Assay

A commercially available fluorometry‐based assay (MAK039, Sigma–Aldrich, Ontario, Canada) was used in this assay. CCa cells under different treatments were collected from 100 mm dish plate after reaching 80% confluency and lysed with lysis buffer. Then, cell lysates were deproteinized by 1 m perchloric acid and neutralized by 3 m potassium bicarbonate solution to make the final pH in the range of 6–8. After diluting, the 50 µL of the sample (≈50 µg total protein) was mixed with 41.8 µL Acetyl‐CoA Assay buffer, 2 µL Acetyl‐CoA substrate mix, 1 µL conversion enzyme, 5 µL Acetyl‐CoA enzyme mix, and 0.2 µL fluorescent probe. For every experiment, a blank control was used to omit the conversion enzyme in the reaction mix. Samples were prepared in a 96‐well plate at 37 °C for 15 min in dark. Fluorescence intensity was measured by using the Thermo Scientific Varioskan LUX at *λ*_ex_ = 535/*λ*_em_ = 587 nm. The results were then normalized to input protein content. Each experiment was repeated three times.

### ATP Concentration, Lactate Generation Assay, and Glucose Uptake

The ATP concentration was calculated using an enhanced ATP assay kit (S0027, Beyotime Biotechnology, Shanghai, China) by measuring chemiluminescence with a luminometer plate reader (Promega Biotech Co. Ltd., Beijing, China) according to the manufacturer's instruction. For lactate generation assay, Lactate Assay Kit‐WST (*λ* = 450 nm; Dojindo) was used following the manufacturer's instruction. For the glucose uptake assay, the Glucose Uptake‐Glo Assay kit (Promega, J1341) was used as described by the manufacturer's instruction. The luminescence was recorded using a GloMaxinstrument by selecting the “Glucose Uptake‐Glo protocol.”

### Oxygen Consumption Rate (OCR)

The Mito Stress Test Kit (Agilent, Cat. No.: 103015–100) was used to measure the OCR following the manufacturer's instructions as previously reported.^[^ ^30^ ^]^ Briefly, before the assay, the probe plate was hydrated with HPLC‐grade water in a CO_2_‐free incubator. The phenol red‐free test solution containing 10 mm glucose, 1 mm pyruvate, 2 mm glutamine, and 5 mm HEPES was placed in a CO_2_‐free incubator at 37 °C to maintain the pH value. The HPLC‐grade water in the hydration plate was then replaced with the calibration solution and stored in a CO_2_‐free incubator at 37 °C. CCa cells were seeded into XF96 cell culture microplates (Seahorse Bioscience) at the density of 10 000 cells/well and kept in an incubator at 37 °C with 5% CO_2_ to adhere to the plate overnight. Oligomycin, FCCP, and Rot/AA were added according to the manufacturer's instructions, respectively (Seahorse Bioscience, North Billerica, MA, USA). Finally, OCR was determined and analyzed on Agilent's Seahorse Bioscience XF96 Extracellular Flux Analyzer (Agilent Technologies) according to the manufacturer's instructions (Seahorse Bioscience, North Billerica, MA, USA).

### Palmitate Uptake

To assess the palmitate uptake ability between HeLa LNM2, SiHa LNM2, HeLa PR, and SiHa PR cells, 2 µCi mL^−1^ (66.67 nm final concentration)[9,10‐3H(N)] palmitate (Perkin Elmer) was incubated for 30 min before washing five times with PBS, followed by cell lysis with 0.1 N NaOH. Radioactivity for radioisotope tracer studies was identified by liquid scintillation counting.

### Statistical Analysis

GraphPad Prism Version 9.0 was used to construct graphs and perform statistical analyses in this study. Two‐tailed Student's *t*‐test was used to analyze differences between groups. Overall survival (OS) was analyzed with the Kaplan–Meier method and calculated by log‐rank test. The relationship between CPT1A expression and the clinicopathological parameters was tested by the χ^2^ test and Fisher's exact test. Correlations were analyzed by using Pearson's correlation. Error bars represent mean ± SD. The difference was considered statistically significant with ^*^ *p* < 0.05, ^**^ *p* < 0.01, and ^***^ *p* < 0.001. Reproducibility was ensured by performing at least three independent experiments unless otherwise specified in the figure captions.

### Ethics Statement

The studies using human tissue samples were approved by the Ethics Committee of The First Affiliated Hospital of Sun Yat‐sen University (Number: IIT‐2022‐205). Animal experiments were approved by the Animal Ethical and Welfare Committee of Sun Yat‐sen University (Number: SYSU‐IACUC‐2022‐001362) and were performed with the provisions of the Declaration of Helsinki of 1975.

## Conflict of Interest

The authors declare no conflict of interest.

## Authors’ Contributions

L.Y., H.J., and Y.J. contributed equally to this work. C.Z., L.Y., C.P., and S.Y. participated in the study design. C.Z., L.Y., H.J., and Y.J. conducted the in vitro and in vivo experiments. YD.L., Y.J., J.L., X.J., C.S., and X.Z. performed the data analyses, Y.Z., Y.L., L.C., H.H., Y.P., W.Wen., W.Wang., and H.J. collected the tissue specimens, C.Z., L.Y., and S.Y. wrote the manuscript. All authors have read and approved of the final manuscript.

## Supporting information

Supporting Information

## Data Availability

Data sharing is not applicable to this article as no new data were created or analyzed in this study.
