# Supplementary material for: Fatty Acid Oxidation Supports Lymph Node Metastasis of Cervical Cancer via Acetyl‐CoA‐Mediated Stemness
Source: Adv Sci (Weinh). 2024 Mar 23;11(21):2308422. doi: 10.1002/advs.202308422 (PMC11151054; doi:10.1002/advs.202308422)
Supplement: Supplementary file 1 — Supporting Information [file ADVS-11-2308422-s001.pdf]

ADVANCED SCIENCE
Open Access

# Supporting Information

for *Adv. Sci.*, DOI 10.1002/advs.202308422

Fatty Acid Oxidation Supports Lymph Node Metastasis of Cervical Cancer via Acetyl-CoA-Mediated Stemness

*Li Yuan, Hongye Jiang, Yan Jia, Yuandong Liao, Caixia Shao, Yijia Zhou, Jiaying Li, Yan Liao, Hua Huang, Yuwen Pan, Weijia Wen, Xueyuan Zhao, Linna Chen, Xu Jing, Chaoyun Pan, Wei Wang*, Shuzhong Yao* and Chunyu Zhang**

## Supporting Information

**Figure S1**

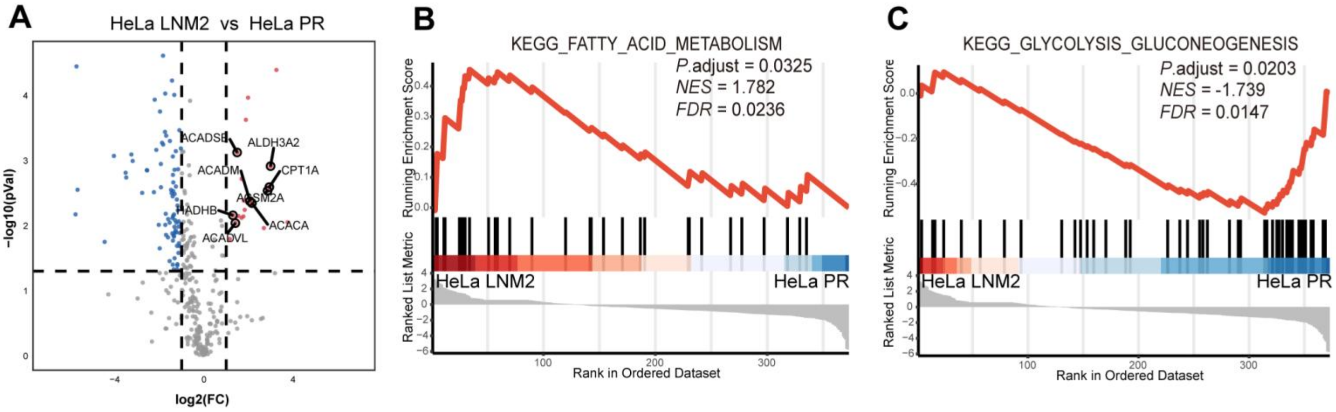


**Figure S1 Metastatic cervical cancer cells in lymph node displayed increased fatty acid oxidation.** A. Differentially expressed transcripts of HeLa LNM2 cells compared with HeLa PR cells involved in cell metabolism were depicted in a volcano plot. The black dotted lines indicated a fold-change threshold of 2, and the *P* value cutoff (*P* = 0.05). B, C. Gene set enrichment analyses of pathways in Figure S1A. The fatty acid metabolism pathway was significantly activated while glycolysis-gluconeogenesis pathway showed an opposite effect.

## Figure S2

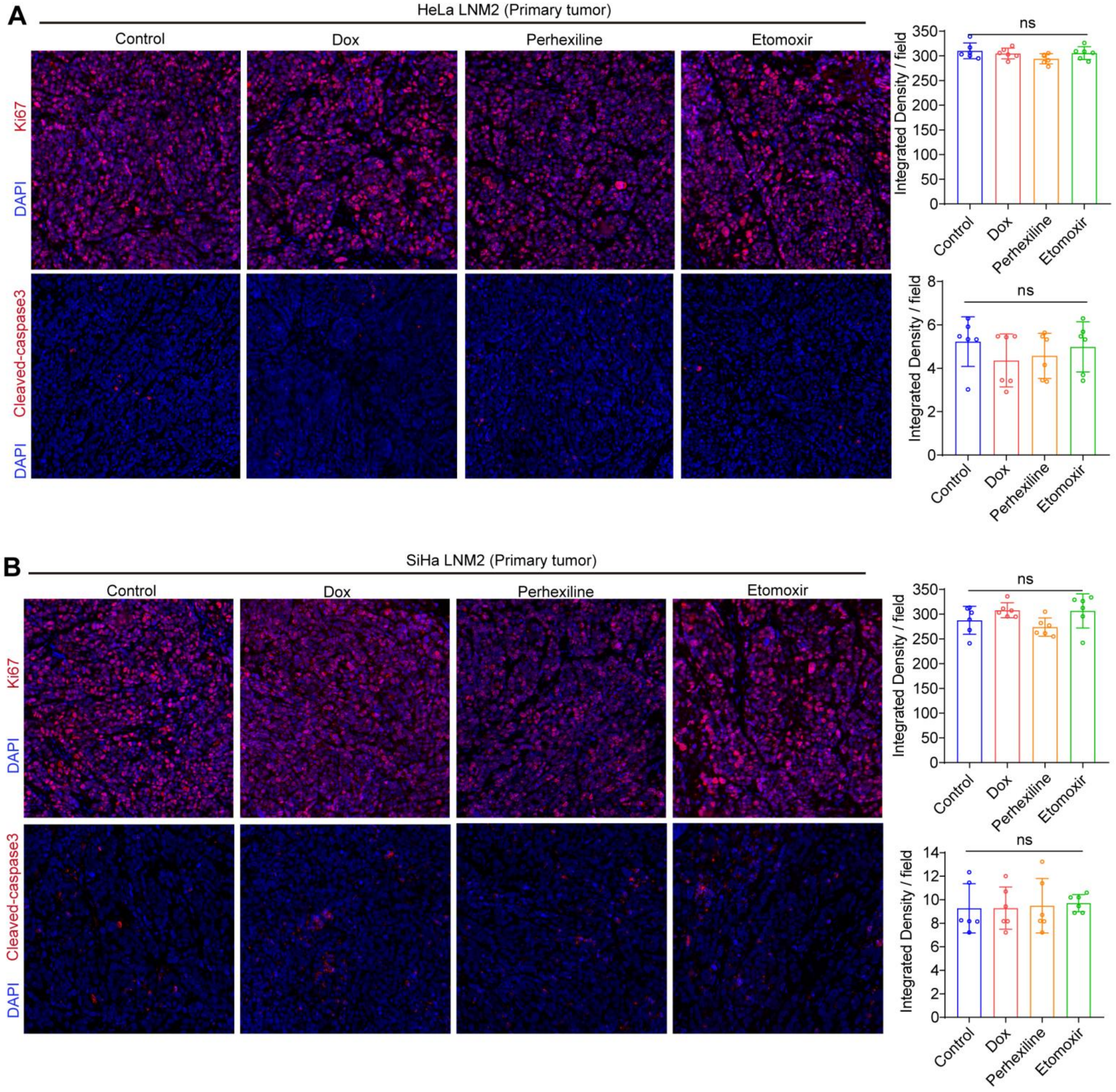


**Figure S2 Inhibition of CPT1A had no significant effect on primary tumor growth.**

A. Representative images of immunofluorescence staining of Ki67 and Cleaved-caspase3 after footpad injection of HeLa LNM2 cells under different treatments (n = 6). B. Representative images of immunofluorescence staining of Ki67 and Cleaved-caspase3 after footpad injection of SiHa LNM2 cells under different treatments (n = 6).Error bars represent the means ± SD. Statistical analyses were performed using a two-tailed Student's t-test. ns, no significance.

## Figure S3

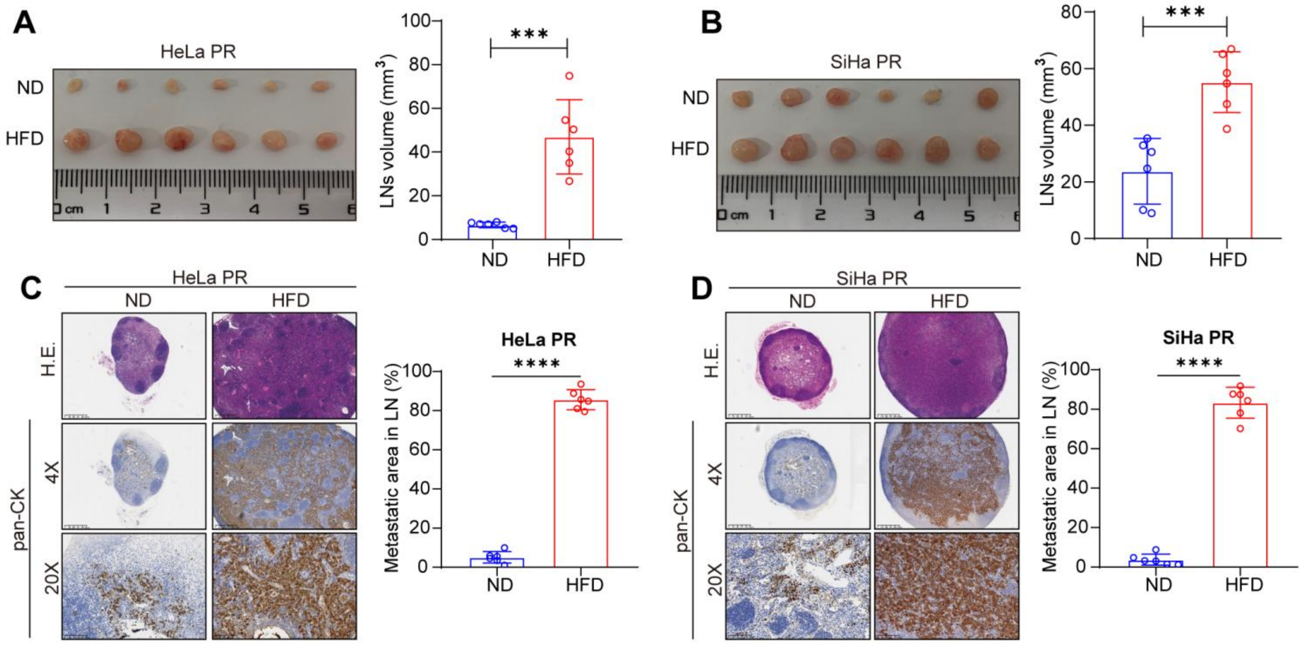


**Figure S3 High-fat diet promoted lymph node metastasis of CCa.** A, B. Representative images and the LNs volume in different groups (n = 6). C, D. Representative images of immunohistochemistry staining of pan-cytokeratin and metastatic area in popliteal LNs. Error bars represent the means ± SD. Statistical analyses were performed using a two-tailed Student's t-test. ***$P < 0.001$; ****$P < 0.0001$.

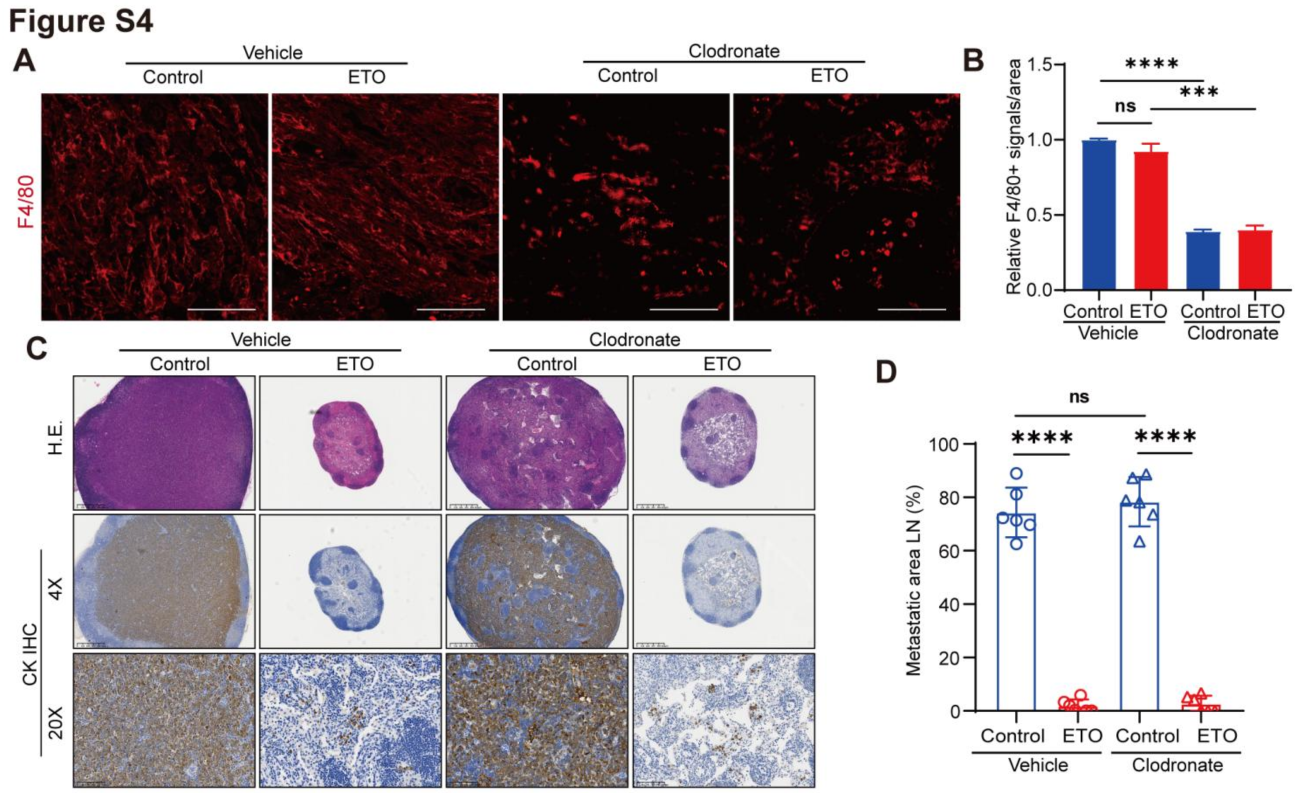


**Figure S4 Depletion of macrophages had no significant influence on LNM of CCa.** A. Representative Immunofluorescence micrographs of F4/80+ macrophage cells in vehicle- and clodronate-treated CCa tumors. Bar = 50 μm. B. Quantification of F4/80+ signals. C. Representative HE and immunohistochemical micrographs of lymph nodes in vehicle- and clodronate-treated groups (n = 6). D. Metastatic area of lymph nodes in each group (n = 6). Error bars represent the means ± SD. Statistical analyses were performed using a two-tailed Student's t-test. ***P < 0.001; ****P < 0.0001, ns, no significance.

## Figure S5

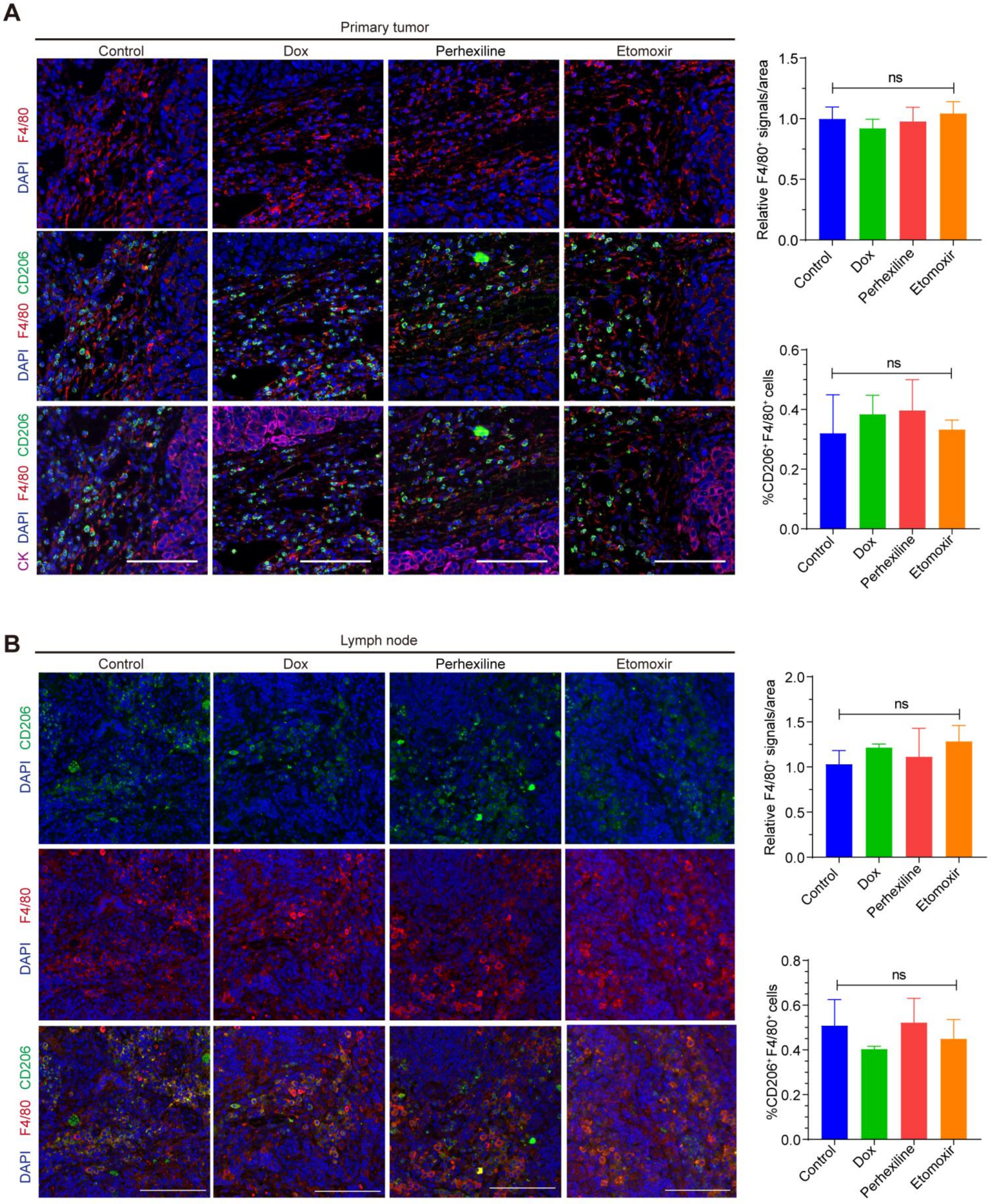


**Figure S5 Inhibition of FAO had no significant influence on macrophage infiltration and polarization.** A, B. Representative immunofluorescent micrographs of total and M2-like macrophages in primary tumor and lymph node under different conditions. Bar = 100μm, and quantification of $F4/80^+$and $CD206^+$ $F4/80^+$signals in the left images. Error bars represent the

means ± SD. Statistical analyses were performed using a two-tailed Student's t-test. ns, no significance.

## Figure S6

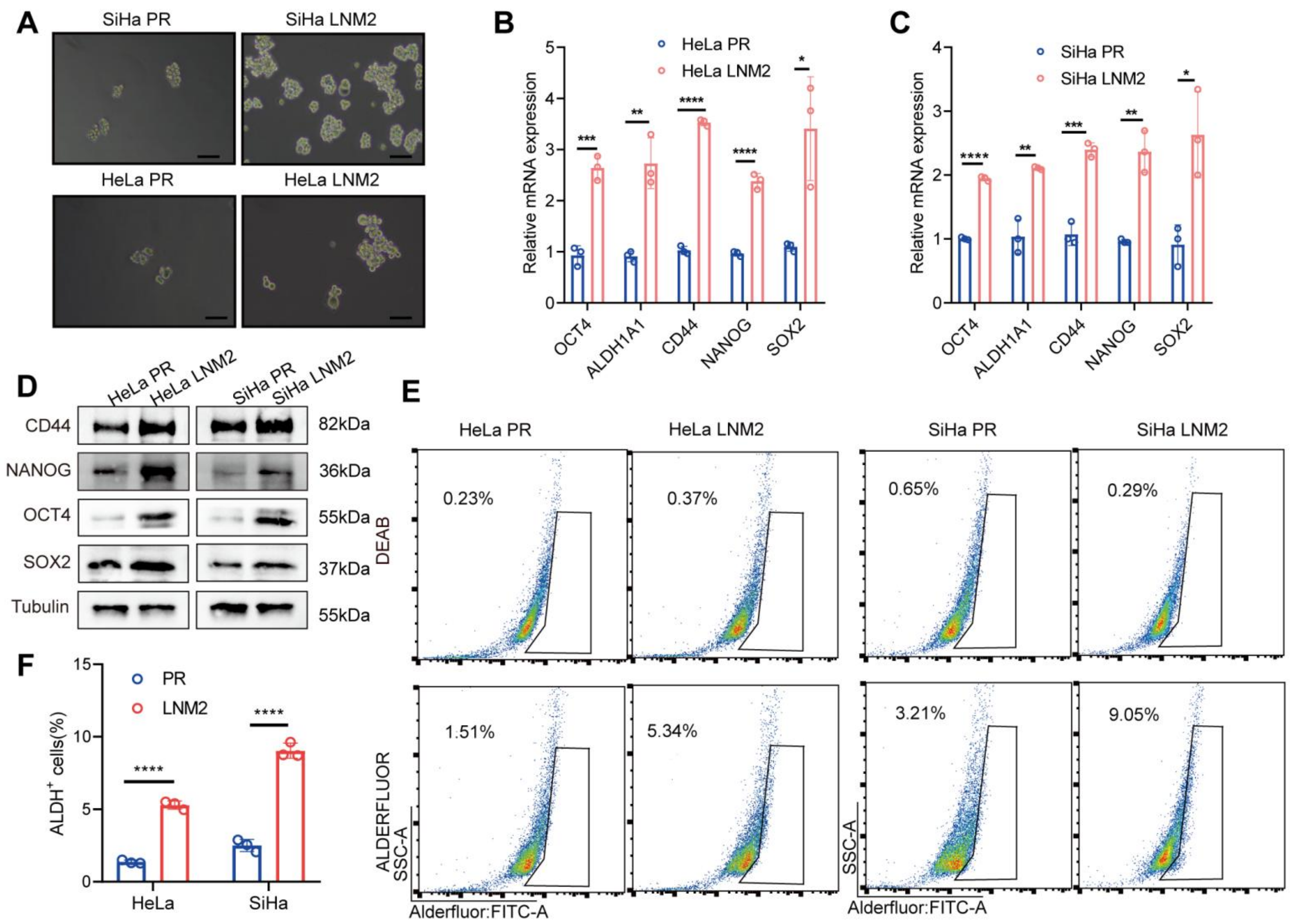


**Figure S6 LN-metastatic cervical cancer cells displayed increased stemness.**

A. Sphere formation assay of HeLa LNM2, SiHa LNM2, HeLa PR, and SiHa PR cells. B, C. The mRNA expression levels of stemness-related genes in CCa cells, Bar = 150μm. D. Relative protein levels of stemness-related genes in CCa cells. E, F. Percentage of ALDH1$^+$ cells detected by flow cytometry. Numerical values are presented as percentage. Each experiment was performed at least three times independently. Error bars represent the means ± SD. Statistical analyses were performed using a two-tailed Student's t-test. *P < 0.05; **P < 0.01; ***P < 0.001; ****P < 0.0001.

**Figure S7**

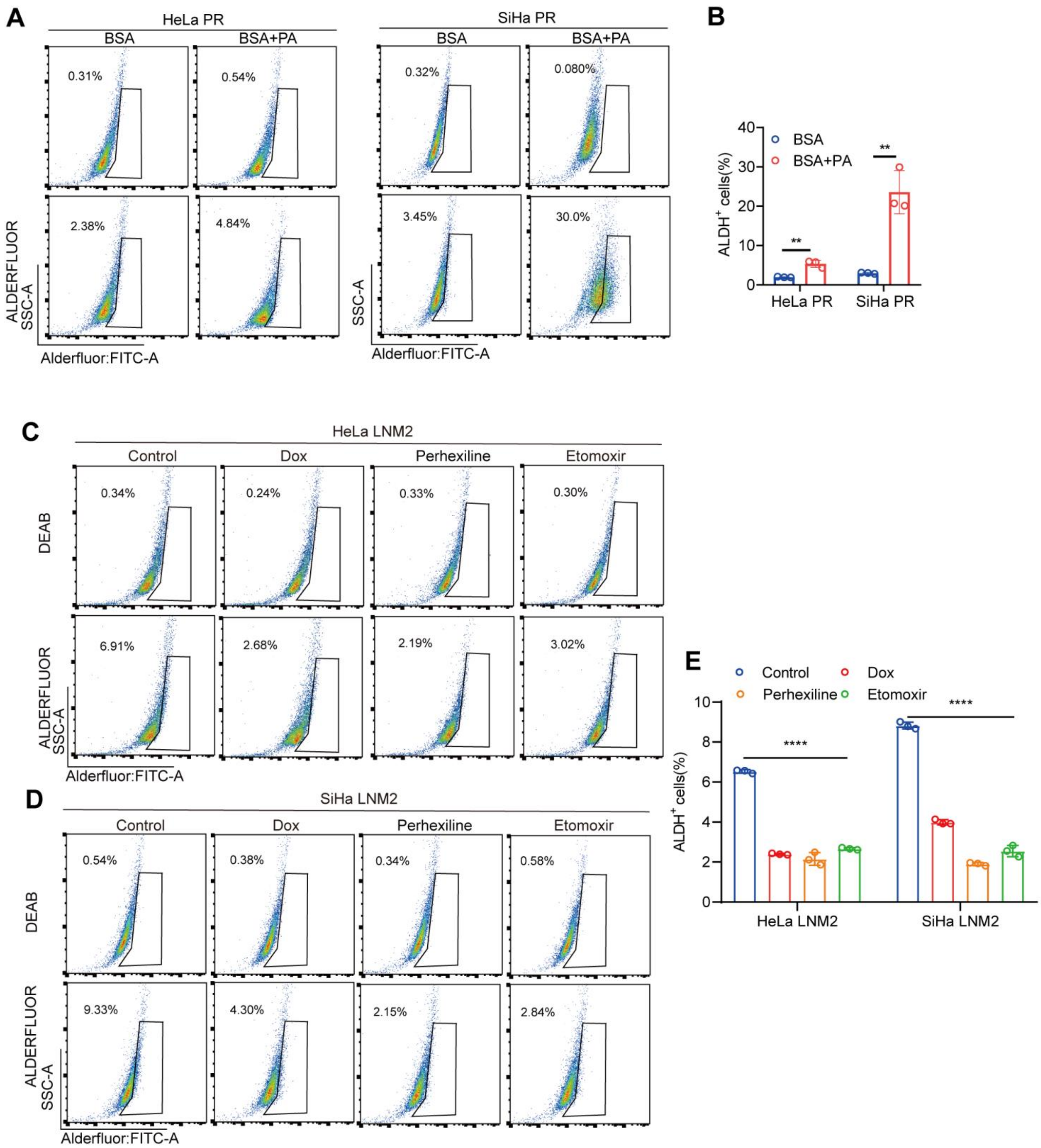


**Figure S7 Fatty acid oxidation participated in the regulation of CCa cells stemness.** A, B. Percentage of ALDH1+ cells detected by flow cytometry in the indicated groups. Numerical values are presented as percentage. C-E. Percentage of ALDH1+ cells detected by flow cytometry in the indicated groups of HeLa LNM2 and SiHa LNM2 cells. Numerical values are presented as percentage. Each experiment was performed at least three times independently. Error bars represent the means ± SD. Statistical analyses were performed using a two-tailed Student's t-test. ****$P < 0.0001$.

Fig. S8

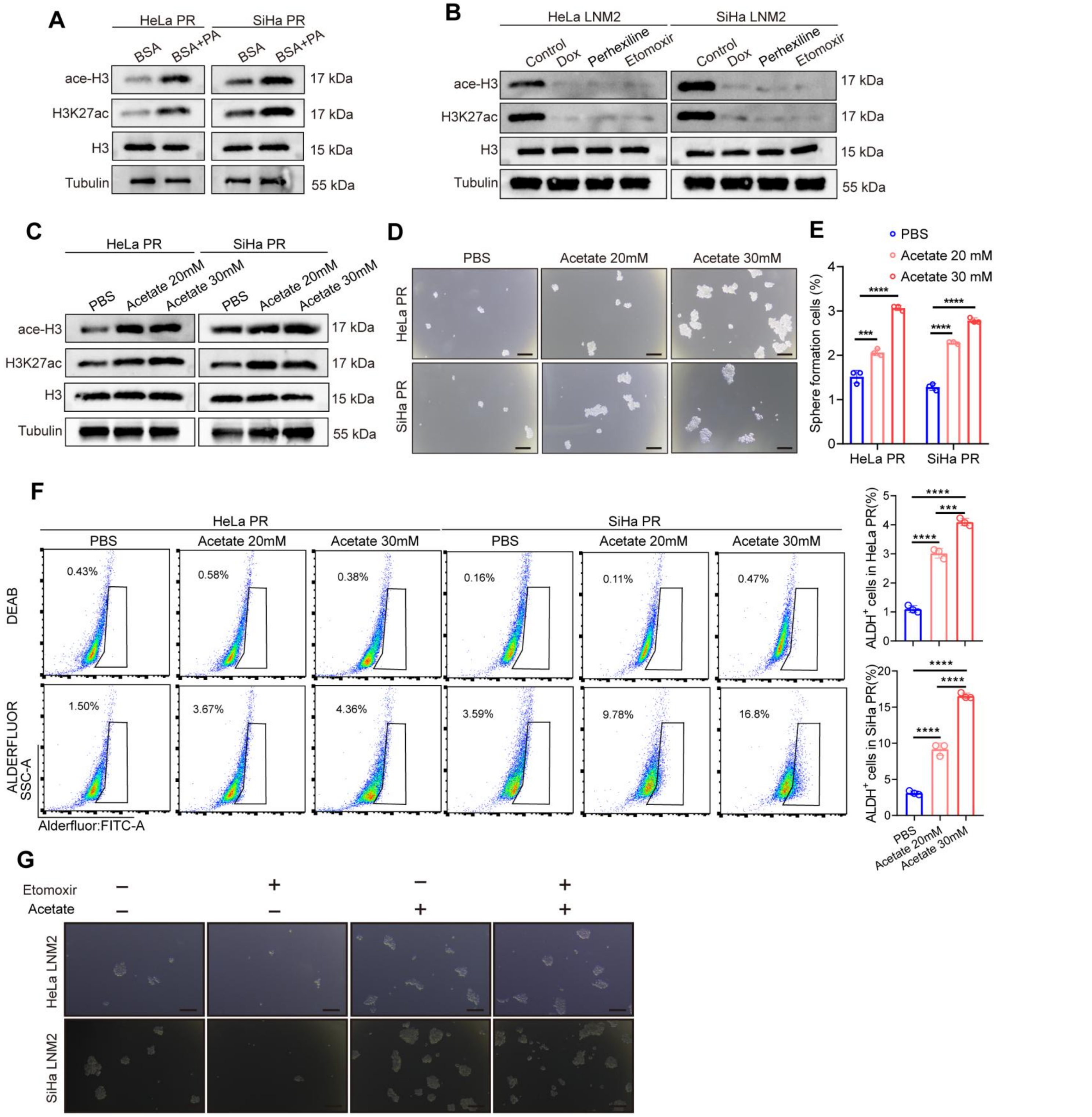


**Figure S8 Fatty acid oxidation derived acetyl-CoA promoted H3K27 acetylation and CCa cells stemness.** A. The expression levels of total acetyl-H3 and H3K27ac were analyzed by Western blotting in CCa cells treated with BSA or PA. B. The expression levels of total acetyl-H3 and H3K27Ac were analyzed by western blotting in the indicated groups. C. A dose-dependent effect accompanied with acetate concentrations. D, E. Sphere formation and sphere formation rate of CCa cells under different dose of acetate supplement. Bar = 150μm. F. Percentage of ALDH1$^{+}$ cells detected by flow cytometry in CCa cells under different dose of acetate supplement.

Numerical values are presented as percentage. Each experiment was performed at least three times independently. G. Sphere formation of CCa cells under different conditions. Bar =150μm. Error bars represent the means ± SD. Statistical analyses were performed using a two-tailed Student's t-test. ***P < 0.001; ****P < 0.0001.

**Figure S9**

**A**

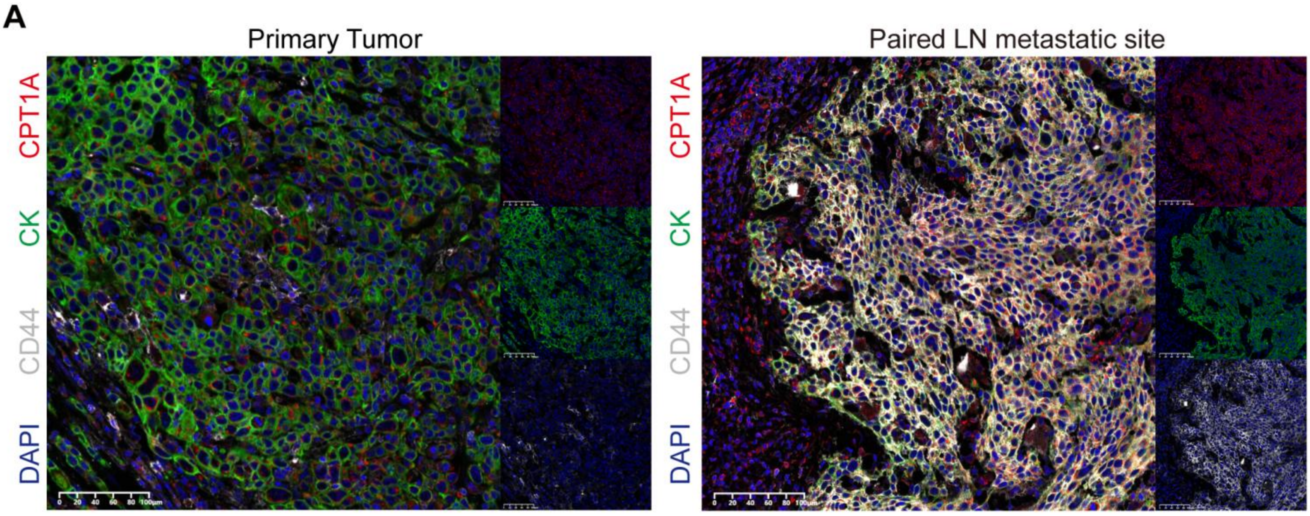


**Figure S9 CPT1A was highly expressed in metastatic CCa cells.**

A. Results of multiple immunofluorescence showed the co-location of CPT1A, CK, and CD44.

**Table S1. Correlation between CPT1A expression with clinicopathological characteristics in 118 cervical cancer patients**

| Variables | Total | CPT1A expression | | *P* value |
|---|---|---|---|---|
| | 118 | Low | High | |
| Age(years) | | 67 | 51 | 0.0726 |
| <42 | 48 | 32 | 16 | |
| ≥42 | 70 | 35 | 35 | |
| FIGO stage | | | | 0.4117 |
| Ⅰ(Ⅰa2+Ⅰb1+Ⅰb2) | 93 | 51 | 42 | |
| Ⅱ(Ⅱa1+Ⅱa2) | 25 | 16 | 9 | |
| Tumor size (cm) | | | | 0.0793 |
| ≤4 | 107 | 64 | 43 | |
| >4 | 11 | 3 | 8 | |
| Pathological types | | | | 0.8094 |
| Squamous cell carcinoma | 104 | 58 | 46 | |
| Adenocarcinoma | 9 | 6 | 3 | |
| Adenosquamous carcinoma | 5 | 3 | 2 | |
| Differentiation | | | | 0.6364 |
| Well | 3 | 2 | 1 | |
| Moderate | 42 | 26 | 16 | |
| Poor | 82 | 39 | 34 | |
| Stromal invasion | | | | 0.3970 |
| <1/2 | 79 | 47 | 32 | |
| ≥1/2 | 39 | 20 | 19 | |
| LVSI | | | | 0.1318 |
| Positive | 17 | 13 | 4 | |
| Negative | 101 | 54 | 47 | |
| LNM | | | | 0.0295 |
| Positive | 34 | 14 | 20 | |
| Negative | 84 | 53 | 31 | |
| Vaginal invasion | | | | 0.4410 |
| Positive | 8 | 3 | 5 | |
| Negative | 110 | 64 | 46 | |
| Parametrial invasion | | | | 0.7089 |
| Positive | 7 | 4 | 3 | |
| Negative | 111 | 63 | 48 | |

**Table S2. Primers used in this study**

| **Primers for RT-qPCR** | | |
|---|---|---|
| ACOX1 | **F** | 5'-ACTCGCAGCCAGCGTTATG-3' |
| | **R** | 5'-AGGGTCAGCGATGCCAAAC-3' |
| ACOX2 | **F** | 5'-GCACCCCGACATAGAGAGC-3' |
| | **R** | 5'-CTGCGGAGTGCAGTGTTCT-3' |
| ACOX3 | **F** | 5'-CTCGATGCCTACCGAGCAAG-3' |
| | **R** | 5'-TGGTTTTCTTAAAGCGGAGCA-3' |
| ACADSB | **F** | 5'-GATGGCAAATGTAGACCCTACC-3' |
| | **R** | 5'-AAGGCCCGGAGTATCACGA-3' |
| ACADVL | **F** | 5'-ACAGATCAGGTGTTCCCATACC-3' |
| | **R** | 5'-CTTGGCGGGATCGTTCACTT-3' |
| ACADM | **F** | 5'-ACAGGGGTTCAGACTGCTATT-3' |
| | **R** | 5'-TCCTCCGTTGGTTATCCACAT-3' |
| ACADL | **F** | 5'-AGGGGATCTGTACTCCGCAG-3' |
| | **R** | 5'-CTCTGTCATTGCTATTGCACCA-3' |
| ACAD8 | **F** | 5'-TCGGAGGGGTCTACATACAAAC-3' |
| | **R** | 5'-CGAAGCTATCAATCATCCAGGCA-3' |
| ACAD9 | **F** | 5'-CCGCCTGTACGAGCTTTCG-3' |
| | **R** | 5'-CACGGGTCCCAAGAACTGAT-3' |
| ACAD10 | **F** | 5'-CAAACACTCGGCCTGTGAAAA-3' |
| | **R** | 5'-ACTAGATCACGATTAGCCAGCC-3' |
| CPT1A | **F** | 5'-TCCAGTTGGCTTATCGTGGTG-3' |
| | **R** | 5'-TCCAGAGTCCGATTGATTTTTGC-3' |
| CPT1B | **F** | 5'-GCGCCCCTTGTTGGATGAT-3' |
| | **R** | 5'-CCACCATGACTTGAGCACCAG-3' |
| CPT1C | **F** | 5'-TTTGCCTCGTGTTTGTGGG-3' |
| | **R** | 5'-CAGCCGTGGTAGGACAGAA-3' |
| ECI2 | **F** | 5'-ATGGGACGCATGGAATGCC-3' |
| | **R** | 5'-TTCAAACCCAGTTGATTTCCTGT-3' |
| EHHADH | **F** | 5'-AAACTCAGACCCGGTTGAAGA-3' |
| | **R** | 5'-TTGCAGAGTCTACGGGATTCT-3' |
| ECHS1 | **F** | 5'-TGAGCTTGCCATGATGTGTGA-3' |
| | **R** | 5'-AACAGGACAAATCTTGCTGACA-3' |
| HADHA | **F** | 5'-ATATGCCGCAATTTTACAGGGT-3' |
| | **R** | 5'-ACCTGCAATAAAGCAGCCTGG-3' |
| HADHB | **F** | 5'-CTGTCCAGACCAAAACGAAGAA-3' |
| | **R** | 5'-CGATGCAACAAACCCGTAAGC-3' |
| LIPE | **F** | 5'-TCAGTGTCTAGGTCAGACTGG-3' |
| | **R** | 5'-AGGCTTCTGTTGGGTATTGGA-3' |
| CD36 | **F** | 5'-AAATAAACCTCCTTGGCCT-3' |
| | **R** | 5'-GCAACAAACATCACCACACC-3' |
| FATP3 | **F** | 5'-CAGAGACCTTCAAACAGCA-3' |
| | **R** | 5'-CAGAACGTACAGTGGTCA-3' |
| FATP4 | **F** | 5'-TCCGCTGGAAAGGTGAGAAC-3' |
| | **R** | 5'-GCATACAGGGGCAGTTCCTT-3' |
| FATP6 | **F** | 5'-GCGTGGTGGCCTTTCTCA-3' |

| | |
|---|---|
| | **R** 5'-ACAGGCGCGGATGCAAT-3' |
| FABP4 | **F** 5'-ACTGGGCCAGGAATTTGACG-3' |
| | **R** 5'-CTCGTGGAAGTGACGCCTT-3' |
| FABP5 | **F** 5'-TGAAGGAGCTAGGAGTGGGAA-3' |
| | **R** 5'-TGCACCATCTGTAAAGTTGCAG-3' |
| OCT4 | **F** 5'-CTGGGTTGATCCTCGGACCT-3' |
| | **R** 5'-CCATCGGAGTTGCTCTCCA-3' |
| SOX2 | **F** 5'-GCCGAGTGGAAACTTTTGTCG-3' |
| | **R** 5'-GGCAGCGTGTACTTATCCTTCT-3' |
| NANOG | **F** 5'-TTTGTGGGCCTGAAGAAAACT-3' |
| | **R** 5'-AGGGCTGTCCTGAATAAGCAG-3' |
| CD44 | **F** 5'-CTGCCGCTTTGCAGGTGTA-3' |
| | **R** 5'-CATTGTGGGCAAGGTGCTATT-3' |
| ALDH1A1 | **F** 5'-GCACGCCAGACTTACCTGTC-3' |
| | **R** 5'-CCTCCTCAGTTGCAGGATTAAAG-3' |
| ACTB | **F** 5'-CATGTACGTTGCTATCCAGGC-3' |
| | **R** 5'-CTCCTTAATGTCACGCACGAT-3' |
| **Primers for ChIP-qPCR** | |
| NANOG | **F** 5'-CCATTCCTGTTGAACCATATTCCT-3' |
| | **R** 5'-ACCCAGACTAGGTGGGGAAC-3' |
| SOX2 | **F** 5'- GTCCCATCCTCATTTAAG -3' |
| | **R** 5'- GAGAAACCTTTGTATCCC -3' |
| OCT4 | **F** 5'- CTGACAGTACAGGTGAAG -3' |
| | **R** 5'- GGAGGTAGGAATACAGGTA -3' |
| **shRNA sequences** | |
| shCPT1A | GCCATGAAGCTCTTAGACAAA |

**Table S3. Antibodies used in this study**

| | | |
|---|---|---|
| CPT1A | CST, #97361 | WB (1:10000)<br>IHC (1:400)<br>IF (1:400) |
| acetyl-Histone H3 | Affinity, #AF4365 | WB (1:1000) |
| H3K27ac | Active motif. #39133 | WB (1 μg/ml dilution) ChIP (10 μg per ChIP) |
| H3 | Affinity, #BF9211 | WB (1:1000) |
| HRP-conjugated Affinipure Goat Anti-Rabbit IgG(H+L) | Proteintech, #SA00001-2 | WB (1:10000) |
| HRP-conjugated Affinipure Goat Anti-Mouse IgG(H+L) | Proteintech, #SA00001-1 | WB (1:10000) |
| Pan-Cytokeratin | CST, #4545S | IHC (1:400), IF (1:500) |
| Ki67 | Proteintech, #27309-1-AP | IHC (1:2000) |
| Tubulin | Abcam, #ab7291 | WB (1:2000) |
| OCT4 | Proteintech, #11263-1-AP | WB (1:1000)<br>IHC (1:400) |
| SOX2 | CST, #3579 | WB (1:1000)<br>IHC (1:400) |
| NANOG | CST, #8822 | WB (1:1000)<br>IHC (1:500) |
| CD44 | Proteintech,<br><br>#15675-1-AP | WB (1:6000); IHC (1:1000) |
| Donkey anti-Rabbit IgG (H+L) Highly Cross-Adsorbed Secondary Antibody, Alexa Fluor™ 555 | ThermoFisher, #A31572 | IF (1:400) |
| Donkey anti-Mouse IgG (H+L) Highly Cross-Adsorbed Secondary Antibody, Alexa Fluor™ 488 | ThermoFisher, #A21202 | IF (1:400) |
| Donkey anti-Rat IgG (H+L) Highly Cross-Adsorbed Secondary Antibody, Alexa Fluor™ 647 | ThermoFisher, #A78947 | IF (1:400) |
| Donkey anti-Mouse IgG (H+L) Highly Cross-Adsorbed Secondary Antibody, Alexa Fluor™ 555 | ThermoFisher, #A31570 | IF (1:400) |
| Donkey anti-Rabbit IgG (H+L) Highly Cross-Adsorbed Secondary Antibody, Alexa Fluor™ 488 | ThermoFisher, #A21206 | IF (1:400) |
| CD3 | Proteintech, #60181-1-Ig | IF (1:500) |
| F4/80 | Abcam, #AB16911 | IF (1:50) |

| | | |
|---|---|---|
| α-SMA | Proteintech, #67735-1-Ig | IF (1:400) |
| CPT1A | Proteintech, #15184-1-AP | IF (1:50) |
| CD206 | Proteintech, #60143-1-Ig | IF (1:400) |
| CD44 | ThermoFisher, #14-0441-82 | IF (1:500) |
